# Acquisition of a novel restriction modification system regulates genetic flux and gene expression in the hypervirulent and globally disseminated CC17 lineage of group B *Streptococcus*

**DOI:** 10.1093/nar/gkag683

**Published:** 2026-07-08

**Authors:** Joana Alves, Chiara Crestani, Stephanie M Marroquin, Alix B E Johnston, Natalie Ring, Connor Bowen, Mark Reglinski, Kelly S Doran, Ruth N Zadoks, Nicola N Lynskey

**Affiliations:** The Roslin Institute, University of Edinburgh, Easter Bush Campus, Midlothian, Scotland EH25 9RG, United Kingdom; Institut Pasteur–Université Paris Cité, Paris 75015, France; Department of Immunology and Microbiology, University of Colorado Anschutz Medical Campus, Aurora, CO 80045, United States; The Roslin Institute, University of Edinburgh, Easter Bush Campus, Midlothian, Scotland EH25 9RG, United Kingdom; The Roslin Institute, University of Edinburgh, Easter Bush Campus, Midlothian, Scotland EH25 9RG, United Kingdom; The Roslin Institute, University of Edinburgh, Easter Bush Campus, Midlothian, Scotland EH25 9RG, United Kingdom; Division of Molecular Microbiology, School of Life Sciences, University of Dundee, Dundee, Scotland DD1 5EH, United Kingdom; Department of Immunology and Microbiology, University of Colorado Anschutz Medical Campus, Aurora, CO 80045, United States; Sydney School of Veterinary Science, Faculty of Science, University of Sydney, Camden, NSW 2570, Australia; The Roslin Institute, University of Edinburgh, Easter Bush Campus, Midlothian, Scotland EH25 9RG, United Kingdom

## Abstract

DNA methylation is a universal mechanism of epigenetic control that in bacteria can regulate genetic flux and gene expression, contributing to the emergence and success of discrete lineages. The CC17 lineage of the important multi-host pathogen group B *Streptococcus* has emerged as a hypervirulent clone in human neonates, the molecular basis for which remains elusive. In this study we identify a novel type II restriction modification system that is uniquely associated with the CC17 clade. Using *in vitro* and *in vivo* techniques, we show that this system acts as a barrier to genetic exchange, which drives the genetic recalcitrance and low levels of homologous recombination associated with the CC17 clade. Strikingly, the restriction modification system also directly regulates expression of the transcriptional activator NanR and promotes murine vaginal colonization and ascension to the uterus, implicating a role for DNA methylation in promoting persistence at polymicrobial mucosal surfaces. Together, these findings reveal for the first time the importance of restriction modification system activity in defining the lineage structure and virulence potential of group B *Streptococcus*, uncovering a novel mechanism that underpins the global success of the CC17 clade as a major neonatal pathogen.

## Graphical Abstract

**Graphical Abstract:**
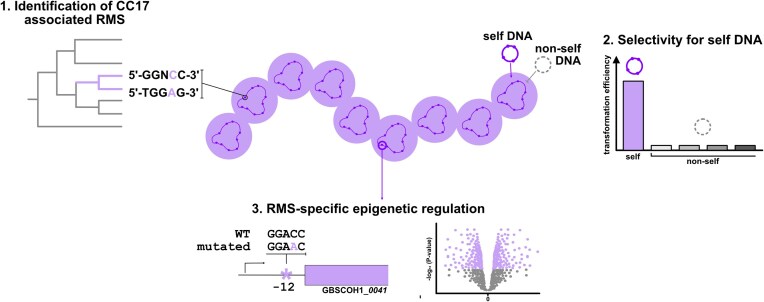

## Introduction

Group B *Streptococcus* (GBS, or *Streptococcus agalactiae*) emerged as a human pathogen in the 20th century, and has since become established as the leading cause of severe invasive infections in neonates worldwide [1]. In humans, GBS is a commensal of the rectovaginal tract that asymptomatically colonizes up to 30% of adults, and presents clinically as a leading cause of bacterial meningitis in neonates, with a case fatality rate of 15% [2]. A single and globally disseminated GBS clone designated CC17 is responsible for over 80% of neonatal meningitis cases [3–5], and is uniquely adapted to the human infection niche.

Despite the global dominance of the CC17 clade among neonatal infections, and the identification of specific bacterial factors such as Srr2 and HvgA associated with the hypervirulence of this clonal lineage [6, 7], the molecular processes that underpin its emergence and success have not been reported. Recent epidemiological studies involving whole genome sequencing of thousands of isolates have unequivocally shown that the evolution of CC17 GBS is distinct from that of all other human disease-associated lineages [3, 5, 8]. The disparity is particularly clear when comparing the rate of recombination within the bacterial genome, which is significantly lower in CC17 GBS (3%) than any other human disease-associated lineage (19%–47%) [9, 10]. This observation raises the possibility that uptake of exogenous DNA, a critical driver of bacterial evolution [11], could be impaired in CC17 strains.

Bacterial uptake of exogenous DNA is tightly regulated to provide protection against viral infection and the acquisition of deleterious genes. This immunity results from the evolution of diverse systems capable of discriminating between self and non-self DNA [12–16], and generates a barrier to the uptake and recombination of exogenous DNA into the bacterial chromosome [17, 18]. Restriction modification systems (RMS) comprise a critical arm of bacterial immunity and are key moderators of genetic flux [19]. Recent studies have identified RMS in >90% of sequenced bacteria across species and genera, highlighting their importance across this domain [20–22].

RMS are divided into four ‘types’ (I–IV) based on structure, and all comprise two key enzymatic domains; a DNA methyltransferase targeting a specific nucleotide motif and a restriction endonuclease that cleaves the same motif when unmethylated [23, 24]. Bacterial strains are frequently associated with multiple RMS, activity of which influences their ability to tolerate foreign DNA. This selective tolerance for exogenous DNA, driven by discrete RMS profiles, defines the flow of genetic information between bacterial lineages and species and plays a critical role in modulating the evolutionary trajectory of important bacterial pathogens [17, 25, 26]. DNA methylation is also a universal mechanism of epigenetic regulation, driving transcriptional variation that can influence the fitness and virulence of clinically important pathogens [21, 27]. Distinct RMS profiles can thus drive variation in genome-wide methylation patterns, generating alternative gene expression profiles and phenotypic heterogeneity with the potential to influence the adaptability, evolution, and clinical importance of bacterial pathogens or lineages [12, 21, 28–32].

Research into the importance of RMS as critical modulators of bacterial evolution and niche adaptation is an emerging field [33]. The distribution of RMS within GBS and their subsequent impact on lineage emergence and structure has not been reported previously. Here, we sought to define the RMS repertoire of GBS and subsequently discern the role of methylation and restriction in the emergence and success of discrete GBS lineages. We show that GBS is associated with multiple RMS, of which two are uniquely associated with the hypervirulent and globally disseminated CC17 clade. Characterization of these RMS revealed that the activity of one, designated SagCOH1II, regulates genetic flux into the CC17 clade. Remarkably, this RMS also enhances transcription of the regulatory gene *nanR_GBS_*, and contributes to vaginal colonization of, and uterine ascension by CC17 GBS. Combined, these data provide novel mechanistic insights into the success of the CC17 clade.

## Materials and methods

### Bacterial strains and growth conditions

GBS strains (Table 1) were cultured on trypticase soy agar supplemented with 5% defibrinated sheep blood (R&D Systems, Minneapolis, Minnesota, USA, Catalog #101114IF) or Todd-Hewitt (TH) (Oxoid, Basingstoke, Hampshire, UK, Catalog #CM189B) agar or in TH broth (THB) at 37°C. Clinical strain COH1, a serotype III ST17 strain isolated from a patient with a neonatal sepsis in 1985 [34], was used as a representative isolate of the CC17 clade and thus used for genetic manipulation. *Escherichia coli* strain NEB5α (New England Biolabs, Ipswich, MA, USA, Catalog #C2987H) was used for storage and passaging of all plasmids and cloning. *Escherichia coli* was cultured on LB agar (Formedium Ltd, Swaffham, UK, Catalog #LMM0202) or broth (Sigma–Aldrich, St. Louis, MO, USA, Catalog #L3022-1KG) at 37°C with shaking at 180 rpm. Growth media were supplemented with antibiotics where appropriate at the following concentrations: for *E. coli*, spectinomycin (Merck, Darmstadt, Germany, Catalog #S4014-5G) at 50 μg/ml and erythromycin (Sigma–Aldrich, St. Louis, MO, USA, Catalog #856 193) at 250 μg/ml; for GBS, spectinomycin (Sigma–Aldrich, St. Louis, MO, USA, Catalog #S4014) at 300 μg/ml and erythromycin (Sigma–Aldrich, St. Louis, MO, USA, Catalog #856 193) at 1 μg/ml.

**Table 1.** Bacterial strains used in this study

Strain	Description	CC	Reference/source
A909	Wildtype serotype 1a	7	[35]
515	Wildtype serotype 1a	23	[36]
BS39	Wildtype serotype 1a		[37]
H36B	Wildtype serotype 1b	6	[35]
SB30	Wildtype serotype 1b		
BS29	Wildtype serotype II		[37]
NCTC10/84	Wildtype serotype V	26	ATCC #49 447
BM110	Wildtype serotype III	17	[38]
COH1	Wildtype serotype III	17	[34]
RI05	Wildtype serotype III (GBS1)	17	Clinical isolate
RI15	Wildtype serotype III (GBS2)	17	Clinical isolate
RI16	Wildtype serotype III (GBS3)	17	Clinical isolate
RI17	Wildtype serotype III (GBS4)	17	Clinical isolate
RI18	Wildtype serotype III (GBS5)	17	Clinical isolate
RI23	Wildtype serotype III (H437)	17	Clinical isolate
RI27	Wildtype serotype III (H730)	17	Clinical isolate
RI28	Wildtype serotype III (H731)	17	Clinical isolate
COH1∆SagCOH1I_RMS	SagCOH1I_RMS deletion mutant	17	This study
COH1∆SagCOH1II_RMS	SagCOH1II_RMS deletion mutant	17	This study
COH1∆SagCOH1II_RMS__REV_	SagCOH1II_RMS revertant	17	This study
GBSCOH1_0041_prom_C:A_	COH1 GBSCOH1_0041 promoter mutant	17	This study
COH1∆*nanR*	*nanR* deletion mutant	17	This study

### Growth curves

Overnight GBS cultures were adjusted to an OD_600_ of 0.05 in THB, ± supplementation with 2 mM sialic acid (N-acetylneuraminic acid, Sigma–Aldrich, St. Louis, MO, USA, Catalog #A0812), in 96-well flat-bottom cell culture plates (Nunc). Plates were incubated at 37°C in a VANTAstar plate reader (BMG Labtech) for 12 h, and OD_600_ measurements recorded every 20 min. Plates were shaken at 300 rpm for 30 s prior to each reading.

### Detection of restriction enzymes in GBS genome assemblies

Restriction enzyme nucleotide sequences of type I, type II, and type III were downloaded from REBASE in October 2023 [39] (https://rebase.neb.com/rebase/rebase.html), and 1254 GBS genome assemblies [10] were screened for their presence with BLAST+ [40] with two subsequent rounds.

A first blast round with strict parametres was carried out to identify significant RMS in the GBS population. Only blast hits with the following characteristics were kept from this first round: (i) 100% of identity and 100% query coverage; (ii) the query start was at position 1 and its name did not contain the word ‘fragment’ (this step ensured that all partial hits, hits with stop codons and enzyme fragments were discarded); (iii) if the subject start and end positions were identical between two hits (i.e. two hits mapped to the same segment of the genome), only the longest hits were kept (this eliminated false positive hits). Twenty-nine enzymes (*n* = 11 of type I, *n* = 16 of type II, and *n* = 2 of type III) were identified after the first blast round as being the most prevalent in the GBS population, as they were detected in ≥15 genome assemblies.

In the second blast round, genome assemblies were screened uniquely for these 29 enzymes. To reduce false positive results, as some enzyme sequences in the database can be very similar to each other, only hits with the highest percentage of identity were kept when two hits mapped to the same region of the genome (i.e. they had the same start and end position). To ensure only complete coding sequences were reported, a maximum of 5% length variation of the subject sequence compared to the reference query was allowed.

### Detection of *nanR* in GBS genome assemblies

Genome assemblies were scanned for *nanR* gene presence with blast+ v2.17.0 (e-value 0.00001) with the reference sequence from the COH1 genome (RefSeq: WP_000158185.1).

### Phylogenetic and recombination analyses of publicly available GBS genomes

IQ-TREE v.2.0.6 [41] was used to infer a core genome phylogeny with a GTR model, from a recombination-free alignment file of 1030 publicly available GBS genome assemblies [10] with Snippy v4.4.5 (https://github.com/tseemann/snippy) and Gubbins v3.2.0 [42], using NGBS128 as reference. Recombination detected with Gubbins was visualized with Phandango [43]. Information on clonal group (CG) number and adaptation to human or animal host species was retrieved from Crestani *et al.* [10]. This analysis excluded animal-restricted CG from the original 1254 sequences to improve clarity, retaining 1030 GBS genomes, which did not affect the interpretation with regards to recombination levels or the presence of RMS.

### Plasmid purification

#### Purification of plasmids from *E. coli*

Plasmid DNA was purified from *E. coli* using a QIAprep Spin Miniprep kit (QIAGEN, Hilden, Germany, Catalog #27104) according to manufacturer’s guidelines.

#### Purification of GBS self-methylated plasmid

Plasmid DNA was purified from successfully transformed GBS strains (see the next section) using a modified QIAprep Spin Miniprep purification protocol as described previously [44, 45]. Briefly, GBS were cultured overnight in 50 ml THB and pellets resuspended in 1 ml QIAprep buffer P1 supplemented with mutanolysin (100 units/ml, Sigma–Aldrich, St. Louis, MO, USA, Catalog #M9901-10KU) and lysozyme (1 mg/ml, Sigma–Aldrich, St. Louis, MO, USA, Catalog #62970-1G-F), and incubated for 30 min at 37°C. Lysates were divided into 4× 250 μl aliquots and mixed with buffers P2 and N3 as per manufacturer’s guidelines. Following a 10-min centrifugation step, supernatants were concentrated two-fold and then purified twice over sequential QIAprep columns. Plasmid DNA was eluted in 50 μl nuclease-free water/column.

#### Transformation of Group B *Streptococcus*

Transformation of GBS was performed as described previously [44, 45]. Briefly, GBS were cultured to OD_600_ 0.2 in 40 ml THB and pellets washed five times in 1 ml ice-cold 0.5 M sucrose (16 000 × *g*, 1 min, 4°C). Pellets were re-suspended in a final volume of 100 μl ice-cold 0.5 M sucrose and 50 μl aliquots were used immediately for electroporation [MicroPulser Electroporator, Bio-Rad; settings: 200 Ω, 1.7 kV, 50 μF, 0.1 cm cuvette (Thermo Fisher Scientific, Waltham, MA, USA, Catalog #732-1135)].

### PCR screen for CC17 RMS

Genomic DNA was extracted from stationary phase GBS cultures as described previously [46], and polymerase chain reaction (PCR) was carried out using a MyCycler (Bio-Rad) thermal cycler with DreamTaq mastermix (Thermo Fisher Scientific, Waltham, MA, USA, Catalog #K1081). SagCOH1I and SagCOH1II were amplified using primers SagCOH1I_F+R and SagCOH1II_KO_1+2 (Table 2), respectively.

**Table 2.** Primers used in this study

Primer name	Sequence 5′-3′
SagCOH1I_F	CACCATCCTGGATGGTACAG
SagCOH1I_R	GACGATAATGCAATCAATGAT
SagCOH1I_KO_1	CG**GGATCC**CGAACAGATTTGAACATCC
SagCOH1I_KO_2	CCTTCTAACATTAGCCTTTCAAAGTCTACTC
SagCOH1I_KO_3	GAGTAGACTTTGAAAGGCTAATGTTAGAAGG
SagCOH1I_KO_4	CGCC**GTCGAC**GATAGTCTGTATACTGTAG
SagCOH1II_KO_1	CG**GGATCC**CTATCGTTGATAACGCACC
SagCOH1II_KO_2	CATCAAATACTGTATCGCTATCAAATTCGAC
SagCOH1II_KO_3	GTCGAATTTGATAGCGATACAGTATTTGATG
SagCOH1II_KO_4	CGCC**GTCGAC**CATATCATCTGGCACTGAAC
nanR_KO_1	CG**GGATCC**CCGCTAGCGCATCATCAGG
nanR_KO_2	CTACTCGATTTTCATAATGCACGGTCCTCT
nanR_KO_3	AGAGGACCGTGCATTATGAAAATCGAGTAG
nanR_KO_4	CGCC**GTCGAC**GTTTTCCAATTGATTGATATC
nanR_GBS__prom_F	CG**GGATCC**CCGCTAGCGCATCATCAGG
nanR_GBS__prom_R	CGCC**GTCGAC**TTAGCTCCGTGACAACTTGC
nanR_GBS__prom_C:A__SDM_1	AAAGAGGAGAGGAACGTGCATTATG
nanR_GBS__prom_C:A__SDM_2	AAGAAAGCGCTTAACATATTCAG
1160_co_transcript_F	GAACCTTTACCAACTGGCTTCA
1160_co_transcript_R	CAAGTCAGTCGTGTCTATGTGG
gyrA_F	AGCACAAAAAACGTGGAGGAC
gyrA_R	ACGATAGGGAGGCCTTTAGC
nanR_GBS__F	CTGGGAGTGATTTGTGAGGC
nanR_GBS__R	TTAGCTCCGTGACAACTTGC
1160_F	GGCTGACCCAAGTAGAGATTTG
1160_R	TGGCACTGAACTTCCACCAG
*nanE*_F	TCATGTCAAGCTTTGCCTGG
*nanE*_R	CTTATACCAACCGCTCCTGC

### Allelic-exchange mutagenesis

Temperature-sensitive *E. coli*–streptococci shuttle vector pJRS233 [47] was used to facilitate generation of isogenic COH1 mutants.

### Generation of GBS deletion mutants

Plasmids pJRS_∆SagCOH1I, pJRS_∆SagCOH1II, and pJRS_∆nanR were generated to facilitate the generation of isogenic deletion mutants COH1∆SagCOH1I_RMS, COH1∆SagCOH1II_RMS, and COH1∆nanR using primers SagCOH1_KO_1–4, SagCOH1II_KO_1–4, and nanR_KO_1–4 (Table 2), respectively. Briefly, 500 bp fragments flanking each RMS were amplified and spliced by overlap extension (SOEing) PCR, incorporating the restriction sites BamHI and SalI into the resulting PCR product to facilitate cloning into pJRS233. The resulting shuttle vectors pJRS_∆SagCOH1I, pJRS_∆SagCOH1II, and pJRS_∆nanR were transformed into strain COH1 by electroporation as described above, and the spliced flanking regions swapped with the RMS genes by allelic exchange mutagenesis. PCR using primers outside of the cloned region was performed to confirm deletion of each RMS.

### Generation of control revertant strain COH∆SagCOH1II_RMS__REV_

pJRS∆SagCOH1II transformants were selected at the permissive temperature (30°C) under antibiotic selection to maintain the plasmid. To promote chromosomal integration via homologous recombination (single crossover), transformants were shifted to the non-permissive temperature (37°C) while maintaining antibiotic selection. Integrants were subsequently passaged at 30°C without antibiotic selection to facilitate a second recombination event (double crossover), resulting in excision of the plasmid backbone and either restoration of the wild-type allele (revertant) or generation of the desired deletion mutant. A revertant generated at the same passage as COH1∆SagCOH1II_RMS was named COH1∆SagCOH1II_RMS__REV_ and used as a control.

### Generation of COH1_GBSCOH1_0041_prom_C9A_

Plasmid pJRS_GBSCOH1_0041_prom_C9A_ was generated to facilitate the generation of an isogenic COH1 strain with an single nucleotide polymorphism (SNP) in the *rpiR* promoter at nucleotide −9. Primers GBSCOH1_0041_prom_F+R (Table 2) were used to amplify a single product comprising 500 bp regions up- and downstream of the *rpiR* promoter, incorporating the restriction sites BamHI (Thermo Fisher Scientific, Waltham, MA, USA, Catalog #FD0054) and SalI (Thermo Fisher Scientific, Waltham, MA, USA, Catalog #FD0644) into the resulting PCR product to facilitate cloning into pJRS233. The resulting plasmid was modified by site directed mutagenesis using the Q5 Site-Directed Mutagenesis Kit (New England Biolabs, Ipswich, MA, USA, Catalog #E0552S) with primers GBSCOH1_0041_prom_C:A__SDM_1+2 (Table 2) to mutate nucleotide C to A at site −9 upstream of the ATG and transformed into strain COH1 by electroporation as described above. The mutated GBSCOH1_0041 promoter was swapped into the chromosome by allelic exchange mutagenesis, which was confirmed by PCR and Sanger sequencing.

### Detection of modified motifs by Nanopore sequencing

Nanopore sequencing was used to identify the DNA sequence motifs being modified in three strains; COH1, COH1∆SagCOH1II_RMS, and COH1∆SagCOH1II_RMS. gDNA was purified from stationary growth phase cultures using the Wizard® HMW DNA Extraction Kit (Promega, Madison, WI, USA, Catalog #A2920) according to manufacturer’s guidelines, and modified to include RNase treatment with RNase Cocktail Enzyme Mix (Thermo Fisher Scientific, Waltham, MA, USA, Catalog #AM2286). The DNA was further purified using the ProNex Size-Selective Purification System (Promega, Madison, WI, USA, Catalog #A2920) according to manufacturer’s instructions with the following modifications: 50 µl gDNA and 200 µl ProNex beads (1:4 ratio) (Promega, Madison, WI, USA, Catalog #NG2001) were used and eluted into 20 µl nuclease-free water, which was incubated at 50°C with 400 rpm mixing on a thermomixer for 10 min.

Purified DNA was quantified using the Qubit dsDNA BR Assay Kit (Thermo Fisher Scientific, Waltham, MA, USA, Catalog #Q32850) and diluted with nuclease-free water to achieve a mass of 200–250 ng in 10 µl. Sequencing libraries were prepared using the Rapid Barcoding Kit (Oxford Nanopore Technologies, Oxford, UK, Catalog #RBK-114.24) and the samples were sequenced on an R10.4.1 flow cell (Oxford Nanopore Technologies, Oxford, UK, Catalog #FLO-MIN114) for 72 h using a GridION device.

All data processing steps can be reproduced using the instructions on our project GitHub page: https://github.com/nataliering/Strep_methylation/. Briefly, the raw pod5 files were basecalled using Dorado v1.0.2 in super accurate, methylation-aware mode (sup,4mC_5mC,6mA) (https://github.com/nanoporetech/dorado). fastq reads were extracted from the resulting .bam file for each sample using samtools v1.13 [48], and genomes assembled using Flye v2.9.6-b1802 [49] in nano-hq mode, setting the expected genome size to ‘2m’. Samtools was used to map, sort, and index the methylated reads from the .bam file to the assembled genome. Modkit v0.4.5 (https://github.com/nanoporetech/modkit) was used to create a pileup of modified reads in .bed format. Finally, Nanomotif v0.6.2 [50] was used in motif_discovery mode to identify modification motifs for each sample.

### Digestion of GBS genomic DNA to visualize DNA methylation

GBS gDNA was purified as described above, and incubated with relevant restriction enzymes [GsuI: 5′-CTGGAG-3′ (Thermo Fisher Scientific, Waltham, MA, USA, Catalog #FD0464); Cfr13I: 5′-GGNCC-3′ (Thermo Fisher Scientific, Waltham, MA, USA, Catalog #FD0194)] at 37°C, 1 h. Samples were visualized in the genomic DNA ScreenTape using the Tapestation System (Agilent Technologies, Santa Clara, CA, USA).

### Cell culture

The VK2/E6E7 (ATCC, Manassas, VA, USA, Catalog/strain #CRL-2616) human vaginal epithelial cell line was maintained in keratinocyte serum free media (KSFM) supplemented with 0.1 ng/ml human recombinant EGF, 0.05 mg/ml bovine pituitary extract (Thermo Fisher Scientific, Waltham, MA, USA, Catalog #17005042), and 0.4 mM calcium chloride at 37°C, 5% CO_2_.

### GBS adherence and invasion assays

VK2/E6E7 cells were seeded in 96-well plates at 7.5 × 10^4^ cells/well 24 h prior to infection. GBS were cultured in THB overnight, washed in KSFM media, and inoculated into each well at an MOI of 100 in the presence or absence of 2 mM sialic acid (N-acetylneuraminic acid). Adhesion assays were carried out following a 4-h incubation at 37°C, 5% CO2. Cells were washed three times with phosphate buffered saline (PBS) to remove non-adherent organisms. Cell-associated bacteria were released by vigorous pipetting following lysis of the cell monolayer with 0.1% Triton X-100, and quantified by serial dilution and plating onto TH agar plates. Adherent bacteria were defined as cell-associated internalized bacteria (quantified as described below).

For invasion assays, VK2/E6E7 cells were infected with GBS as described for adherence assays, but prior to lysis, the cells were incubated with KSFM containing 100 μg/ml gentamicin (Sigma–Aldrich, St. Louis, MO, USA, Catalog #G1397) and 5 µg/ml penicillin G (Sigma–Aldrich, St. Louis, MO, USA, Catalog #P3032) at 37°C for 30 min to kill residual adherent GBS. Cells were then washed three times with PBS to remove residual antibiotics, and internalized bacteria released by lysis of the cell monolayer with 0.1% Triton X-100, and quantified by serial dilution and plating onto TH agar plates.

For RNA extraction, VK2/E6E7 cells were infected as described above for adherence assays. Cells were washed three times with PBS and stored at −80°C in TRI reagent (Zymo Research, Irvine, CA, USA, Catalog #R2071) prior to RNA extraction. Bacterial inocula were cultured in cell culture plates at 37°C, 5% CO_2_ in KFSM in the presence or absence of 2 mM sialic acid (N-acetylneuraminic acid, Sigma) concurrently. Bacteria were harvested, centrifuged at 4000 × *g* for 5 min, the pellet suspended in TRI reagent (Zymo Research, Irvine, CA, USA Catalog #R2050), and stored at −80°C prior to RNA extractions.

### RNA extraction

GBS strains were cultured in THB to OD_600_ 0.4 (logarithmic phase) or 0.9 (stationary phase), and pellets re-suspended in 300 µl of TRI reagent (Zymo Research, Irvine, CA, USA, Catalog #R2071). Bacterial lysates were prepared by bead beating (pulsed 3× for 30 s) in ice-cold 2 ml Precellys Lysing tubes (CK01, Bertin Corp, Montigny-le-Bretonneux, France, Catalog #CK01) using a Precellys 24 tissue homogenizer (Bertin Corp, Montigny-le-Bretonneux, France). RNA was extracted using the Direct-zol RNA Miniprep Plus Kit (Zymo Research, Irvine, CA, USA, Catalog #R2071) according to manufacturer’s guidelines and eluted in 50 µl of RNAse/DNase-free water. An additional genomic DNA removal step was performed using TURBO DNA-free Kit (Thermo Fisher Scientific, Waltham, MA, USA, Catalog #AM1907). Total RNA was quantified using Qubit Broad Range RNA BR Assay Kit (Thermo Fisher Scientific, Waltham, MA, USA, Catalog #Q10210) according to manufacturer’s guidelines.

### Quantitative Real-time PCR

Complementary DNA (cDNA) was synthetized from 100 ng of purified RNA using the RevertAid First Strand cDNA Synthesis Kit (Thermo Fisher Scientific, Waltham, MA, USA, Catalog #K1621), and an RT negative control prepared for each sample. Quantitative real-time PCR (qRT-PCR) was performed using SYBR Green Brilliant II qPCR Master Mix (Agilent Technologies, Santa Clara, CA, USA, Catalog #600828) on an AriaMx real time PCR thermocycler (Agilent Technologies, Santa Clara, CA, USA). qRT-PCR was carried out on GBSCOH1_0041 and GBSCOH1_1160 (primer pairs nanR_GBS__F+R, 1160_F, and *nanE*_F+R, respectively), and expression data normalized to that of *gyrA* (primer pair gyrA_F+R) using the ΔΔct method. All primer sequences are provided in Table 2.

### RNA-sequencing and data analysis

RNA-sequencing, including library preparation, was performed by Novogene using the Illumina NovaSeq X Plus Sequencing System platform to generate 150 bp paired-end reads. Data were analysed using the Galaxy Europe server (https://usegalaxy.eu). Trimmomatic (Galaxy Version 0.39+galaxy2) was used to process raw reads, remove adapter sequences, and perform quality control. Clean reads were aligned to the annotated GBS COH1 genome (NCBI Genbank accession number: HG939456.1) using HISAT2 (Galaxy Version 2.2.1+galaxy1). Transcripts were quantified using featureCounts (Galaxy Version 2.0.3+galaxy2), and differential gene expression analysis was performed using DESeq2 (Galaxy Version 2.11.40.8+galaxy0).

### Co-transcription analysis of SagCOH1II

Co-transcription analysis was performed by PCR using cDNA and gDNA purified from COH1 and COH1∆SagCOH1II_RMS as described previously, using primers 1160_co_transcript_F+R (Table 2).

### Protein structural predictions

Predicted protein structures were generated using AlphaFold2 hosted on Google Colab [51]. Structural homologues were identified using the FoldSeek server [52]. Structural representations and superpositions were generated using the PyMOL Molecular Graphics System, Version 2.0 (Schrödinger).

### Murine vaginal colonization model

Seven-week old female CD-1 mice were purchased from Charles River Laboratories. Mice were housed in specific ABSL facilities at the University of Colorado Anschutz Medical Campus (CU-AMC) and allowed to acclimate for 1 week prior to experimentation. All animal work was approved by and performed in accordance with the Institutional Animal Care and Use Committee of the CU-AMC under protocol #00316.

One day prior to colonization, female CD-1 mice were injected intraperitoneally with 0.5 mg β-estradiol in 100 μl sesame oil in order to synchronize their estrous cycles. Mice were vaginally lavaged with 100 μl of sterile PBS by gently pipetting 50 μl of sterile PBS (approximately eight times up and down) and repeating once more with 50 μl of fresh sterile PBS. Vaginal lavage was plated on CHROMagar™ StrepB selective chromogenic media and incubated overnight at 37°C. Any mice positive for GBS following lavage were excluded from the study. Following synchronization, mice were intravaginally inoculated directly with ∼10^7^ CFU of mid-log phase GBS strains COH1 or COH1∆SagCOH1II_RMS in 10 μl per mouse. Following inoculation, mice were vaginally lavaged daily with 100 μl of sterile PBS by gently pipetting 50 μl of sterile PBS (approximately eight times up and down) and repeating once more with 50 μl of fresh sterile PBS. Vaginal lavage was vortexed briefly, serially diluted 10^−1^ through 10^−4^, track-plated on CHROMagar™ StrepB, and incubated overnight at 37°C. Undiluted samples were spot-plated for every day post-inoculation. This was repeated through experimental end-point. Lavage data represent two independent experiments with *n* = 10 mice per group, per experiment.

On day 4 of the second experiment, the reproductive tract of mice was harvested to enumerate for GBS burden. Mice were humanely euthanized by primary CO_2_ and secondary cervical dislocation. Following dissection of the reproductive tract, the vagina, cervix, and uterus were separated and placed into separate 2-ml screw-capped tubes containing ∼1 cm of 1-mm zirconia beads and 500 μl sterile PBS. Tubes were weighed before and after adding tissues to calculate tissue weight. Homogenization was performed by bead beating for 1 min at maximum speed and resting on ice for 1 min for a total of two repetitions. Tissue homogenate was vortexed briefly, serially diluted 10^−1^ through 10^−4^, track-plated on CHROMagar™ StrepB, and incubated overnight at 37°C. Undiluted samples were spot-plated for every tissue. Tissue data are from one independent experiment, *n* = 10 mice per group.

### Statistical analyses

#### Transformation efficiency comparisons

Data represent the mean and standard deviation of four independent experiments. A one-way ANOVA test with multiple comparisons was performed on log-transformed data to identify significant differences.

#### RT-PCR transcription comparisons

Data represent the mean and standard deviation of three independent experiments. A one-way ANOVA test with multiple comparisons was performed to identify significant differences.

#### Association of RMS with specific GBS lineages

A Chi-squared test was used to compare the frequency of each RMS within CC17 with non-CC17 strains, under the null hypothesis that no difference would be observed.

#### Growth curves

Data represent the mean and standard deviation of three independent experiments. A two-way ANOVA test with multiple comparisons was performed to identify significant differences.

#### Murine vaginal colonization

Data were generated from two independent experiments (*n* = 10 mice/group, per experiment), and a two-way ANOVA with uncorrected Fisher’s LSD test performed to compare colonization of mice infected with wild-type COH1 GBS and the isogenic SagCOH1II_RMS deletion mutant.

#### GBS burden in murine tissues

Data were generated from single independent experiment (*n* = 10 mice/group), and an unpaired *t*-test with Welch’s correction used to compare COH1 versus ∆SagCOH1II_RMS.

For all analyses a *P*-value <.05 was deemed significant.

## Results

### CC17 GBS exhibit poor transformation efficiency indicative of low levels of genetic flux

**Figure 1.**
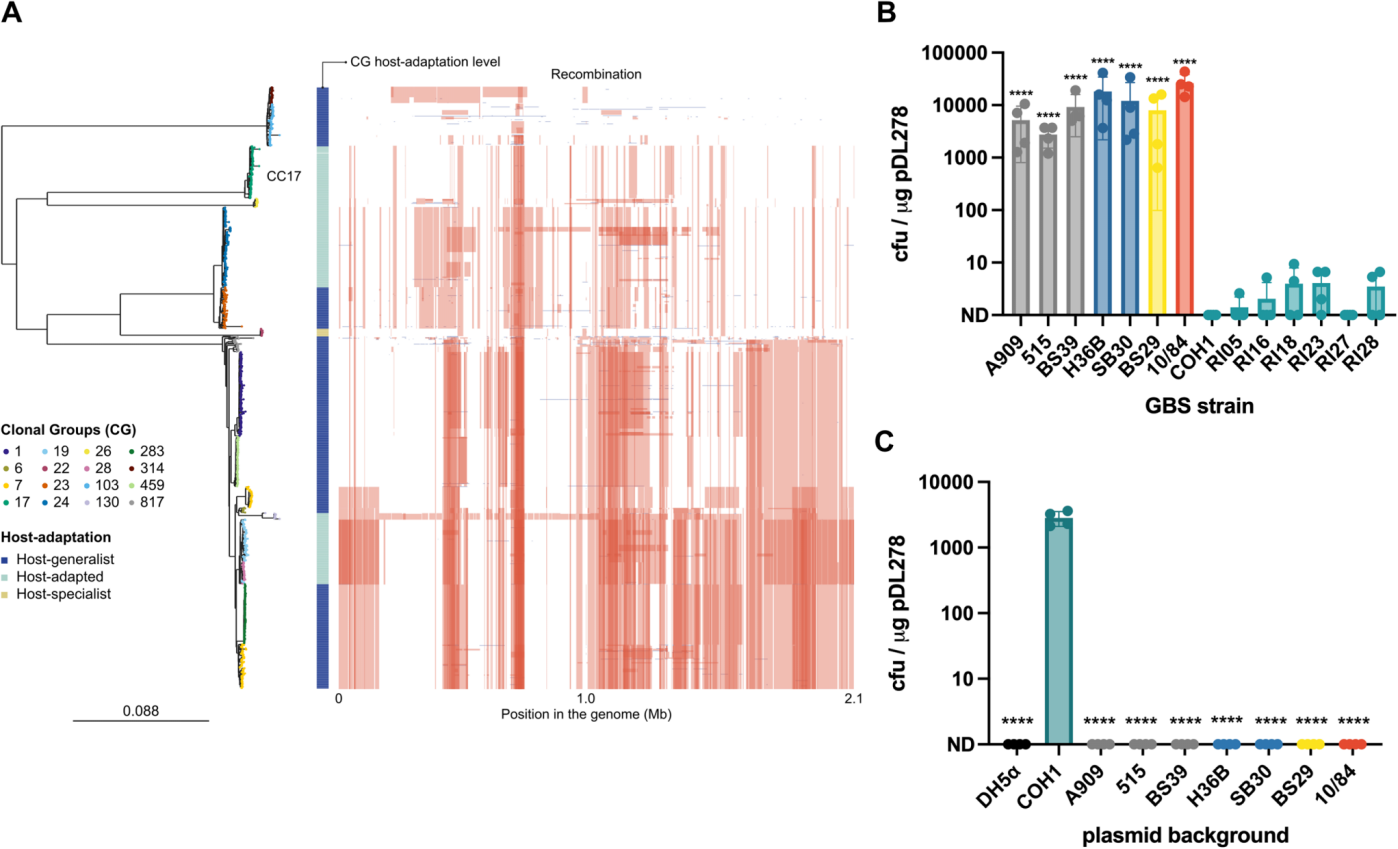
CC17 GBS exhibit poor transformation efficiency indicative of low levels of genetic exchange. (**A**) Maximum-likelihood phylogenetic tree of 1030 GBS core genomes for strains isolated from human, bovine, and piscine hosts (adapted from [10]). Leaf colours indicate CG (an SNP-based system that has superseded the older clonal complex (CC) nomenclature system, which predates widespread availability of whole genome sequences and was based upon related sequence types [10]), and external strips show CG host adaptation level (host generalist <80% from a single host, host adapted 80%–98% from a single host, host specialist ≥98% from a single host [10]), and homologous recombination, highlighting recombination taking place within and between lineages. CC17 (equivalent to CG17) is annotated and shows low levels of recombination. (**B**) Comparison of transformation efficiency of CC17 GBS (green bars, *n* = 7) with representative isolates from the five major human-disease associated serotypes [serotype 1a (grey bars, *n* = 3); serotype II (blue bars, *n* = 2); serotype IV (yellow bars, *n* = 1; and serotype V (orange bars, *n* = 1)] with plasmid pDL278. Transformation efficiency of CC17 GBS is impaired compared with all other strains. Data represent the mean and standard deviation of four independent experiments (one-way ANOVA test with multiple comparisons between each clinical isolate versus all CC17 strains performed on log-transformed data; ****P < .0001). No difference in transformation efficiency was observed between CC17 strains (one-way ANOVA test with multiple comparisons between COH1 versus each CC17 strain performed on log-transformed data; *P *> .05). (**C**) Comparison of transformation efficiency of CC17 strain COH1 with plasmid pDL278 purified from DH5α- *E. coli* (black bar), COH1 (green bar), and each of the strains highlighted in panel (B). Transformation efficiency of COH1 was enhanced with self-methylated plasmid. Data represent the mean and standard deviation of four independent experiments (one-way ANOVA test with multiple comparisons between plasmid purified from each clinical isolate versus COH1 performed on log-transformed data; ****P < .0001).

Homologous recombination is a fundamental driver of bacterial evolution and adaptation, generating genetic diversity through the exchange of short homologous regions of DNA between strains. Rates of recombination differ drastically across species [11] and can also vary significantly amongst discrete lineages within a single species [10, 17]. Recombination events can be quantified within bacterial genomes using the program Gubbins [42] to define the fraction of the bacterial genome that is recombinant, providing insight into the recent phylogenetic history of species and lineages. CC17 GBS exhibit very low levels of recombination (Fig. 1A) [9, 10, 42], the basis and implications of which are unknown. To ascertain whether this could be attributed to an impaired ability to acquire exogenous DNA, transformation efficiency experiments were performed. Uptake of plasmid DNA (pDL278 purified from DH5α *E. coli*) by a panel of CC17 strains was compared with clinical isolates representing the other dominant lineages associated with neonatal infections. The CC17 strains exhibited an extremely poor transformation efficiency, 3 log-fold lower than all other lineages tested with only 0–10 colonies/μg plasmid DNA. Importantly no difference was observed in transformation efficiency between the CC17 strains tested (Fig. 1B).

The plasmid DNA used in these initial experiments was purified from DH5α *E. coli*, and we thus sought to ascertain whether the poor transformation efficiency observed for CC17 strains was due to incompatibility with DNA purified from *E. coli*, or DNA modifications such as methylation. To address this, the clinically relevant neonatal meningitis isolate COH1 [34] was selected as a representative CC17 strain that has been widely used in virulence studies [34, 53–57], and transformed with plasmid DNA purified from each of the GBS strains tested in Fig. 1B, including strain COH1 itself. If the plasmid DNA had been subjected to protective modifications such as methylation within each strain, an increase in transformation efficiency would be observed. Only plasmid DNA purified from the CC17 background showed improved transformation efficiency into COH1 (Fig. 1C). Importantly the same phenotype was also observed for an additional CC17 strain, RI23 (Supplementary Fig. S1). This indicated that strains in this lineage modify DNA in a protective way that facilitates persistence within the bacterium following uptake. We hypothesized that RMS-mediated DNA methylation was contributing to this phenotype.

**Figure 2.**
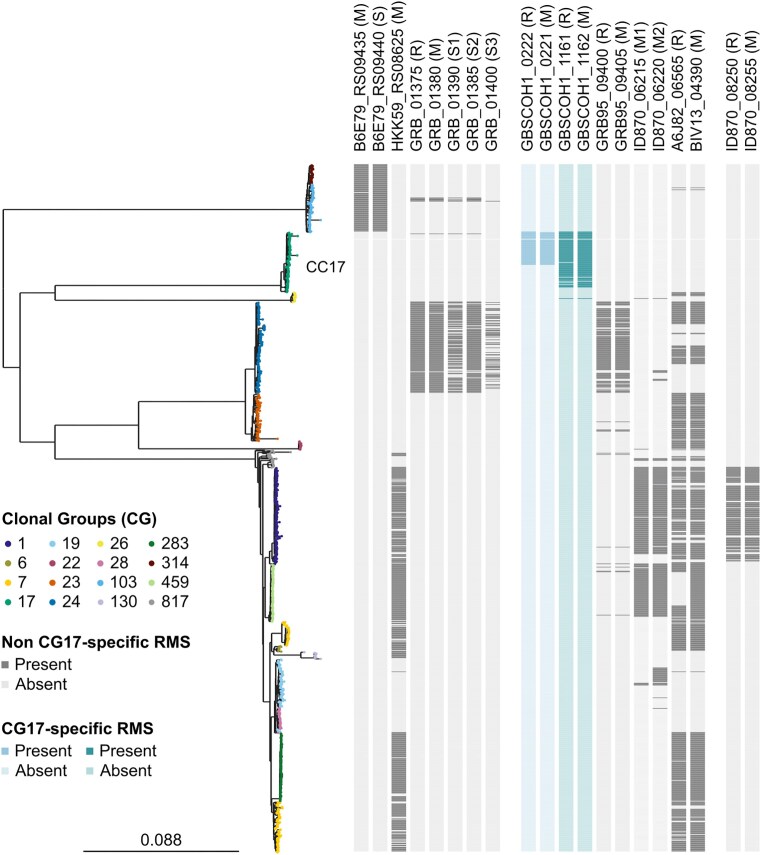
RMS exhibit lineage-dependent distribution within GBS. Maximum-likelihood phylogenetic tree of 1030 GBS core genomes for strains isolated from human, bovine, and piscine hosts. Leaf colours indicate CG, and external strips show the distribution of RMS, grouped by type. Gene names are given from a relevant strain with an available complete genome sequence (in order: BSE009, O1173, 515, COH1, CJB111, HU-GS5823). RMS associated with CC17 GBS are highlighted in colour [SagCOH1I (blue); SagCOH1II (green)]. RMS components are given in parentheses: R = restriction endonuclease; M = methyltransferase; S = specificity protein.

To determine whether RMS activity was responsible for the genetic recalcitrance exhibited by CC17 strains, we sought to identify and characterize the distribution of RMS throughout the GBS population. As such, bioinformatic analyses were performed on a subset of 1030 GBS strains from a previously published collection, representing all lineages and major host species, including humans, cattle, and fishes [10]. Intriguingly, two RMS were uniquely associated with the CC17 lineage (Fig. 2). These observations led us to hypothesize that the activity of one or both systems could be responsible for the genetic recalcitrance we had observed for the CC17 lineage, with a potentially significant impact on the emergence and subsequent pathophysiology of the CC17 clade.

### CC17 GBS are associated with a unique restriction modification system repertoire

**Figure 3.**
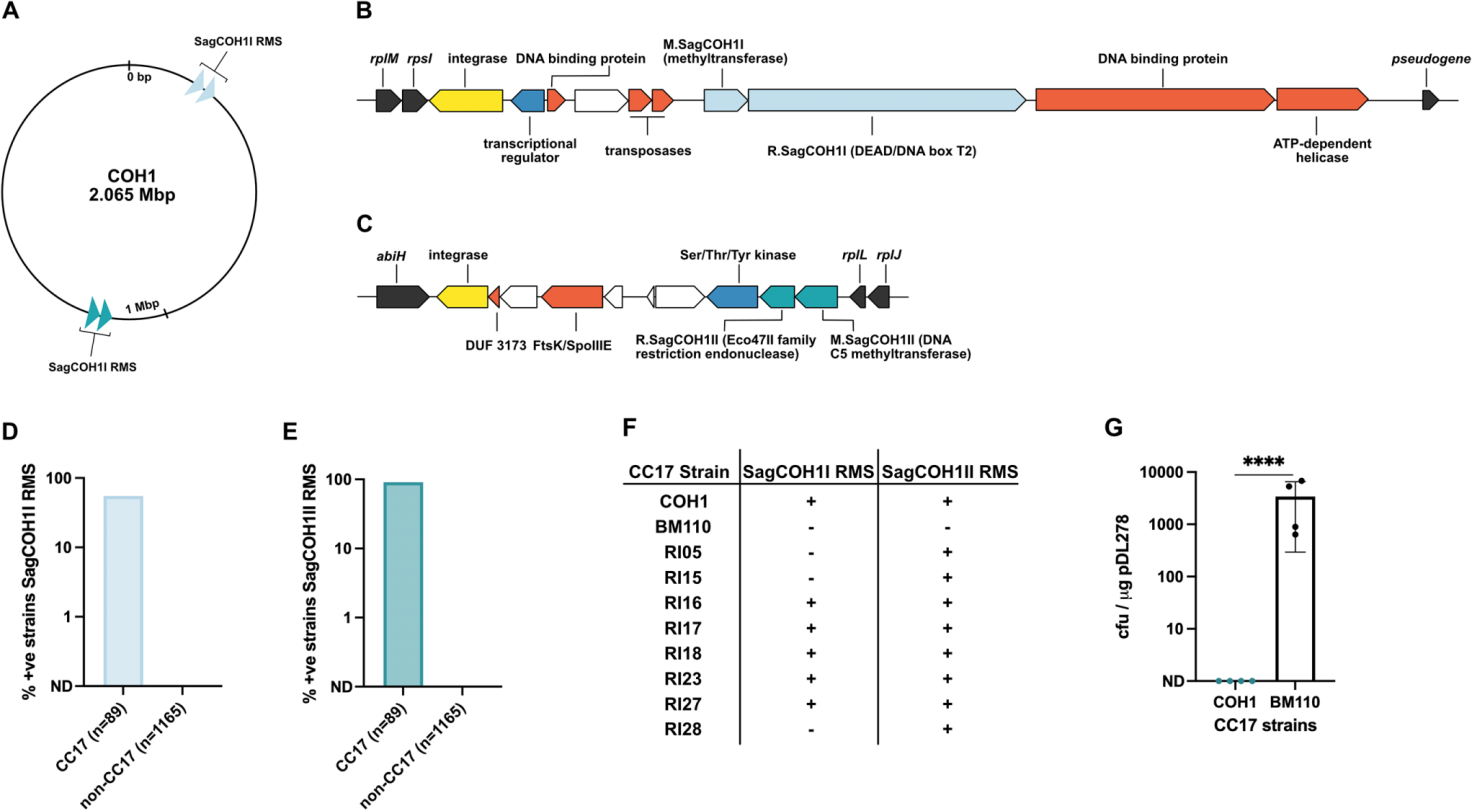
CC17 GBS are associated with a unique RMS repertoire. (**A**) Schematic representation of the COH1 genome and genetic location of each identified RMS [SagCOH1I (blue) and SagCOH1II (green)]. Schematic representation of mobile genetic elements encoding SagCOH1I (**B**) and SagCOH1II (**C**). (**D**) Distribution of SagCOH1I in GBS strains isolated from human, bovine, and piscine hosts (*n* = 1254) [CC17 GBS (filled bar) all other GBS (clear bar)]. SagCOH1II is associated with CC17 GBS [Chi^2^ (1df) = 667.5, *P *< .0001)]. (**E**) Distribution of SagCOH1II in GBS strains isolated from human, bovine, and fish hosts (*n* = 1254) [CC17 GBS (filled bar) all other GBS (clear bar)]. SagCOH1I is associated with CC17 GBS [Chi^2^ (1df) = 1119, *P *< .0001). (**F**) Table depicting the presence/absence of SagCOH1I and SagCOH1II in CC17 strains present in our isolate collection (*n* = 10). (**G**) Comparison of transformation efficiency of CC17 strains COH1 and BM110 (naturally RMS negative) with plasmid pDL278 purified from DH5α *E. coli*. Transformation efficiency of BM110 was significantly higher than that of COH1. Data represent the mean and standard deviation of three independent experiments (multiple unpaired *t*-test analysis on log-transformed data; ****P < .0001).

Using the COH1 genome as a reference, we went on to define the chromosomal location of the two RMS present within CC17 GBS (Fig. 3A) and named the systems based on their gene ID using the REBASE guidelines [39]: SagCOH1I (genes GBSCOH1_0221 and GBSCOH1_0222) and SagCOH1II (genes GBSCOH1_1161 and GBSCOH1_1162). Further, we ascertained that both RMS were encoded on mobile genetic elements (Fig. 3B and C), with insertion sites near (or adjacent to) ribosomal genes, indicating that they were likely to have been acquired by horizontal gene transfer [58].

The specific association of SagCOH1I and SagCOH1II with the CC17 clone was confirmed by Chi-squared analysis (Fig. 3D and E). SagCOH1II was almost ubiquitous amongst the CC17 strains in our genome sequencing collection (91%; *n* = 89; Fig. 3E) compared with the lower prevalence of SagCOH1I (69%; *n* = 89; Fig. 3D). We next screened a panel of CC17 clinical isolates in our strain collection for each RMS. The panel included BM110 [38], a strain that is frequently used as a representative CC17 isolate in studies involving GBS mutagenesis. In line with the population level data (Figs 2 and 3D and E), almost all CC17 strains (7/8) contained SagCOH1II, however only 4/8 encoded SagCOH1I (Fig. 3F). Unexpectedly our screen revealed that BM110 does not encode either RMS and thus serves as a rare and natural CC17 RMS negative control. This also potentially explains its utility for mutagenesis studies [6, 7, 16].

To ascertain whether RMS activity could explain the low levels of DNA uptake exhibited by CC17 GBS, we performed experiments comparing the transformation efficiency of the CC17 strains COH1 (positive for both RMS) and BM110 (negative for both RMS) using plasmid pDL278 purified from DH5α *E. coli*. As expected, COH1 was resistant to transformation, however BM110 had a transformation efficiency equivalent to non-CC17 lineages of GBS (Figs 3G and 1B). These data strongly implicated RMS activity as a key driver of the genetic recalcitrance exhibited by the CC17 clade. We thus set out to functionally characterize CC17-associated RMS to test this hypothesis.

### SagCOH1II regulates genetic flux within CC17 GBS

Genes within both CC17-associated RMS were annotated as having methyltransferase domains, and we sought to confirm this activity by generating isogenic deletion mutants of each RMS in strain COH1 (SagCOH1I mutant: COH1∆SagCOH1I_RMS; SagCOH1II mutant: COH1∆SagCOH1II_RMS), to enable the functional characterization of each system experimentally.

**Figure 4.**
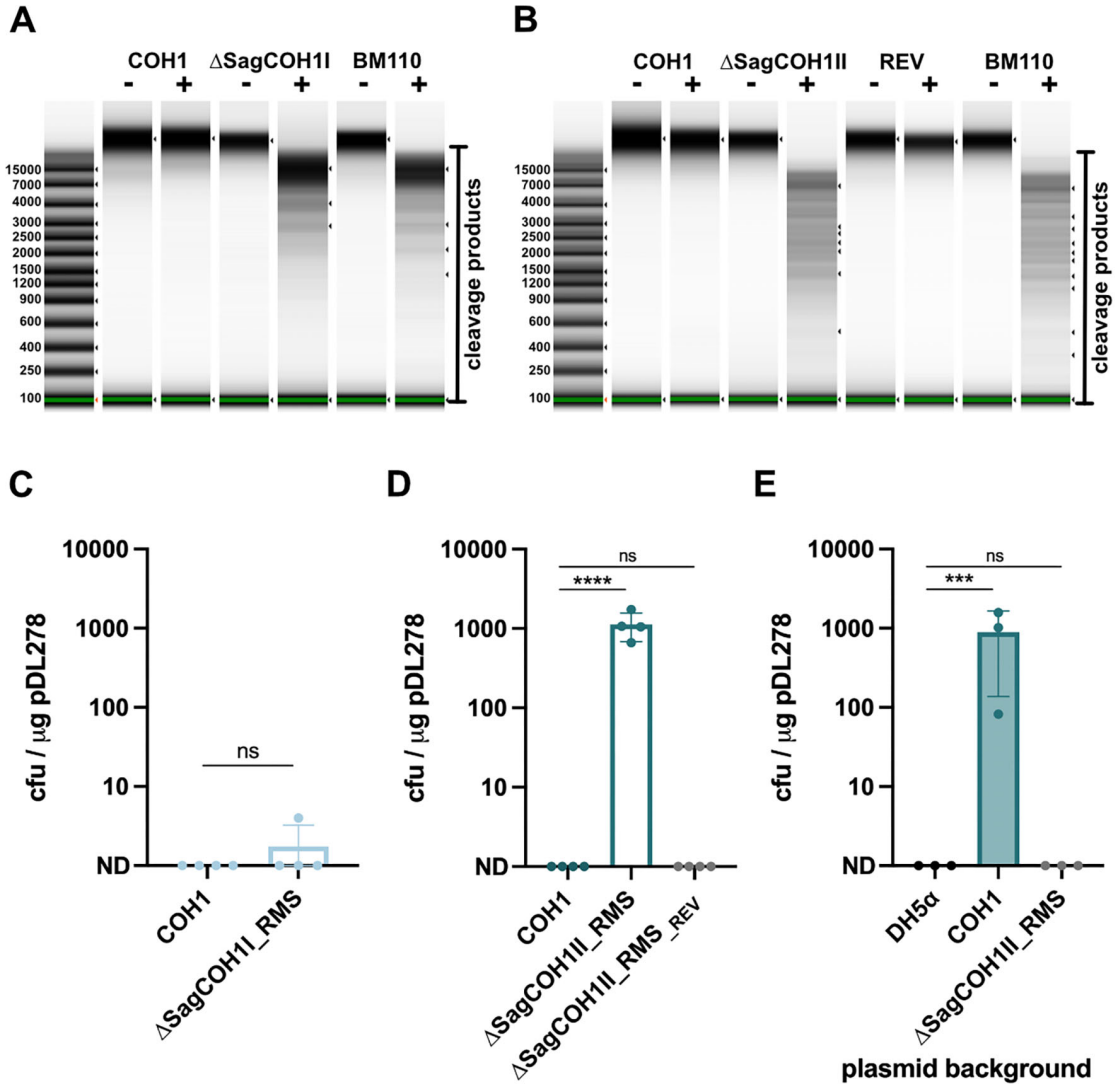
SagCOH1II regulates genetic flux within CC17 GBS. (**A**) SagCOH1I methylation protects CC17 GBS gDNA from cleavage by restriction enzyme GsuI. Deletion of SagCOH1I in COH1 (∆SagCOH1I_RMS) abrogates resistance of gDNA to GsuI cleavage (−, without GsuI; +, with GsuI). BM110 (naturally RMS negative) gDNA is susceptible to cleavage. (**B**) SagCOH1II methylation protects CC17 GBS gDNA from cleavage by Cfr13I. Deletion of SagCOH1II in COH1 (∆SagCOH1II_RMS) abrogates resistance of gDNA to Cfr13I cleavage (−, without Cfr13I; +, with Cfr13I), and is restored in the revertant strain COH1∆SagCOH1II_RMS__REV_. BM110 gDNA is susceptible to cleavage. (**C**) Comparison of transformation efficiency of CC17 strain COH1 (filled blue bar) with deletion mutant ∆SagCOH1I_RMS (empty blue bar) following electroporation with plasmid pDL278. Deletion of SagCOH1I did not impact transformation efficiency. Data represent the mean and standard deviation of three independent experiments (multiple unpaired *t*-test analysis on log-transformed data; ns = *P *> .05). (**D**) Comparison of transformation efficiency of CC17 strain COH1 (filled green bar) with deletion mutant ∆SagCOH1II_RMS (empty green bar) and revertant ∆SagCOH1II_RMS__REV_ (filled grey bar) following electroporation with plasmid pDL278. Deletion of SagCOH1II enhanced transformation efficiency. Data represent the mean and standard deviation of three independent experiments (one-way ANOVA test with multiple comparisons performed on log-transformed data; ****P < .0001). (**E**) Comparison of transformation efficiency of CC17 strain COH1 with plasmids pDL278 purified from DH5α *E. coli* (black bar), COH1 (green bar), and COH1∆SagCOH1II_RMS (grey bar). Transformation efficiency of COH1 was enhanced with self-methylated plasmid where SagCOH1II_RMS was present. Data represent mean and standard deviation of three independent experiments (one-way ANOVA test with multiple comparisons performed on log transformed data; ***P < .001; ns = *P *> .05).

The methyltransferase activity of each RMS was confirmed using Nanopore sequencing, and the target sequence for each system identified using Nanomotif v0.6.2 [50] (SagCOH1I: 6mA, 5′-TGGAG-3′; SagCOH1II: 5mC, 5′-GGNCC-3′). These data were then experimentally validated by performing restriction enzyme cleavage assays on purified gDNA. Methylated wild-type COH1 gDNA was resistant to cleavage by enzymes targeting the same sequence motif as each RMS. Importantly, gDNA purified from each isogenic mutant was susceptible to cleavage by an isoschizomer (SagCOH1I, GsuI; SagCOH1II, Cfr13I of the restriction enzyme encoded by the deleted RMS (Fig. 4A and B). These data demonstrate that both CC17-associated RMS elicit methyltransferase activity that protectively modifies host gDNA.

We next sought to ascertain whether either system was responsible for the poor transformation efficiency associated with CC17 GBS. To quantify the relative contribution of each CC17-associated RMS to the genetic recalcitrance observed for CC17 GBS, transformation efficiency experiments were performed. GBS were transformed with plasmid pDL278, which contains multiple target sites for both CC17-associated RMS (SagCOH1I: 11 sites; SagCOH1II: 12 sites) and thus is susceptible to cleavage by both systems. Only deletion of SagCOH1II was sufficient to restore transformation efficiency to levels observed for other GBS lineages (Fig. 4C and D). Importantly, only plasmid purified from wild-type COH1, and not ∆SagCOH1II_RMS, was associated with a significant increase in transformation efficiency, indicating that activity of SagCOH1II is essential to protectively modify exogenous DNA (Fig. 4E). Combined, these data demonstrate that SagCOH1II, which is uniquely associated with CC17 GBS, is responsible for the genetic recalcitrance of this clonal lineage and likely contributed to the low recombination rates reported for this clade (Fig. 1A).

### SagCOH1II epigenetically regulates gene expression in CC17 GBS

**Figure 5.**
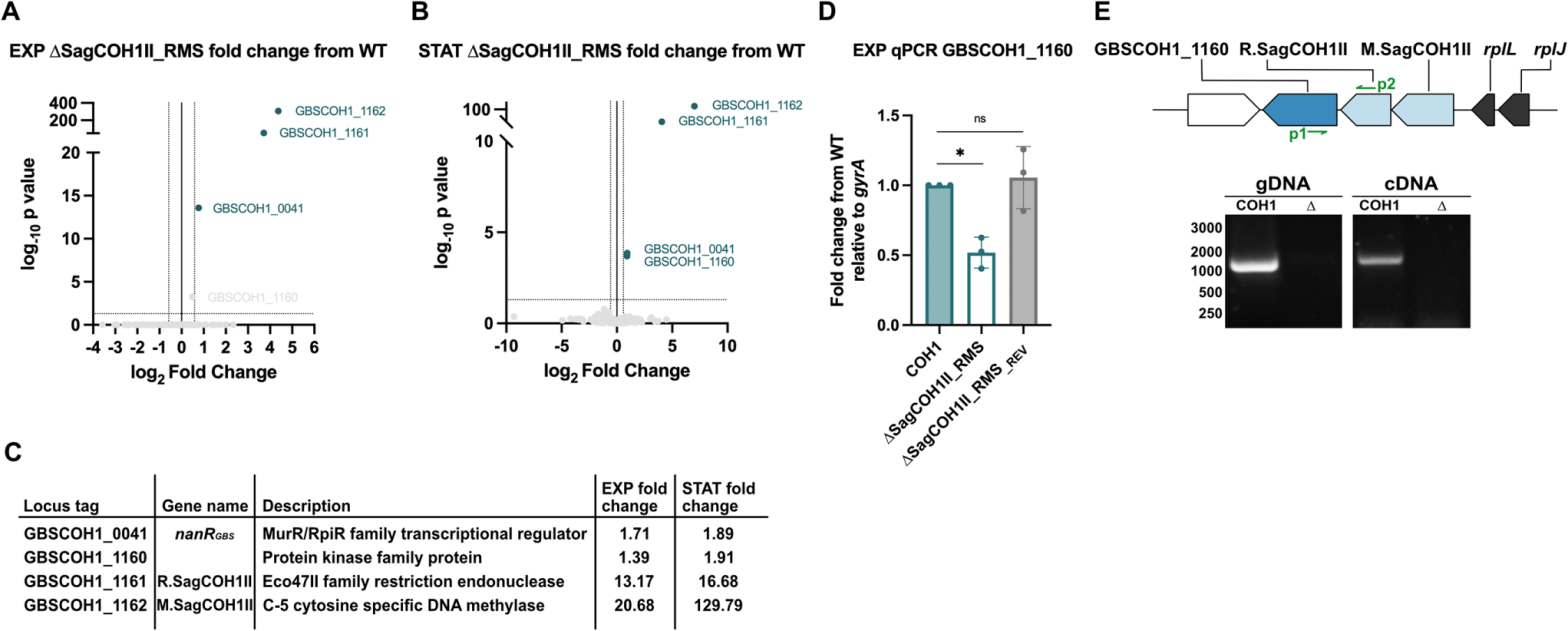
RNAseq of COH and ∆SagCOH1II_RMS. (**A**) Volcano plot showing genes with differential expression in COH1∆SagCOH1II_RMS compared with wild-type COH1 during exponential growth. (**B**) Volcano plot showing genes with differential expression in COH1∆SagCOH1II_RMS compared with wild-type COH1 during stationary growth. (**C**) Table detailing genes differentially expressed in COH1∆SagCOH1II_RMS compared with COH1. (**D**) Quantitative RT-PCR was used to validate differential expression of GBSCOH1_1160 mediated by SagCOH1II_RMS. GBSCOH1_1160 transcription at OD_600_ 0.4 was compared in wild-type COH1 and ∆SagCOH1II_RMS__REV_. SagCOH1II was associated with increased expression of GBSCOH1_1160. Data represent the mean and standard deviation of three independent experiments (one-way ANOVA test with multiple comparisons; *P < .05, ns = *P *> .05). (**E**) Schematic representation of GBSCOH1_1160 and surrounding genes with p1 and p2 highlighting location of primers used to discern whether this gene is co-transcribed with SagCOH1II_RMS. Successful amplification results in generation of a 1000-bp product. Gel depicts product amplification by PCR performed using these primers on gDNA and cDNA purified/generated from COH1 and ∆SagCOH1II_RMS. Generation of an identical 1000-bp product from gDNA and cDNA templates indicates that GBSCOH1_1160 is co-transcribed with R.SagCOH1II (GBSCOH1_1161).

Epigenetic regulation has the potential to alter global transcription patterns with a significant impact on bacterial pathophysiology [21, 28, 29, 31, 32]. To ascertain whether DNA methylation mediated by either SagCOH1I or SagCOH1II could regulate transcription within CC17 GBS, RNAseq was performed. Only deletion of SagCOH1II gave rise to any significant differences in transcription, with a similar pattern of gene expression observed at logarithmic (Fig. 5A) and stationary phases of growth (Fig. 5B and Supplementary Table S1). The impact on transcription was minimal, but showed consistent upregulation of two genes, GBSCOH1_1160 and GBSCOH1_0041 by SagCOH1II (Fig. 5C). To validate and discern the functional significance of these results, each gene was assessed in turn.

The GBSCOH1_1160 gene is located 58 bp upstream of SagCOH1II within the same putative mobile genetic element. We first validated the RNAseq data by reverse transcriptase-polymerase chain reaction (RT-PCR) and included the revertant strain COH1∆SagCOH1_RMS__REV_ as a control (Fig. 5D). Based on the gene ontology, we predicted and were able to prove that GBSCOH1_1160 was co-transcribed with R.SagCOH1II, indicating that the genes form a distinct operon (Fig. 5E). Whether the transcriptional change is due to polar effects following deletion of SagCOH1II or direct regulation is unknown. Importantly, we show that GBSCOH1_1160 is a member of the SagCOH1II operon, the biological significance of which is under investigation.

### SagCOH1II-mediated DNA methylation controls GBSCOH1_0041 transcription

**Figure 6.**
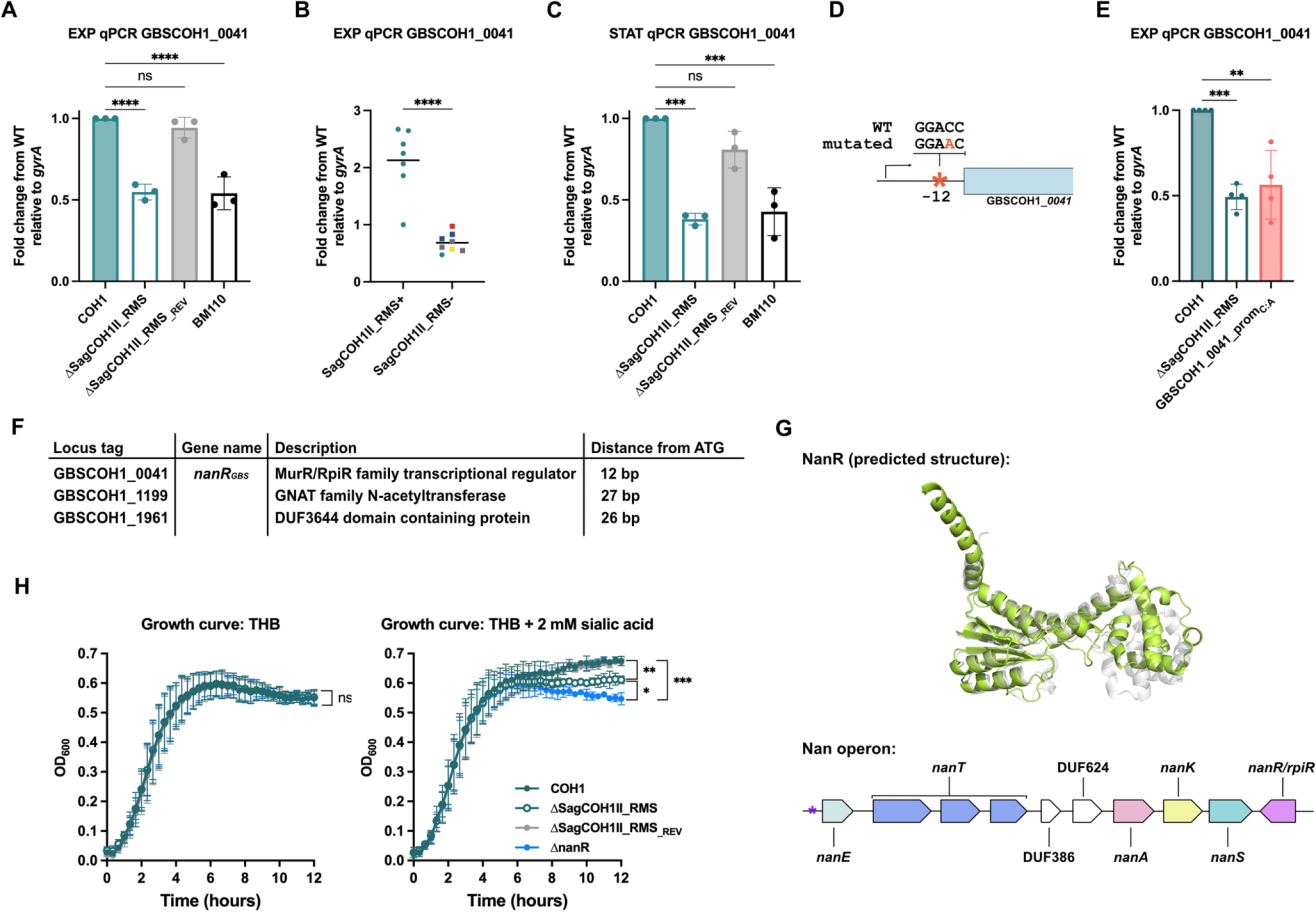
SagCOH1II methylation regulates expression of the transcriptional regulator NanR_GBS_. (**A**) Quantitative RT-PCR was used to validate differential expression of *nanR_GBS_* mediated by SagCOH1II. *nanR_GBS_* transcription at OD_600_ 0.4 was compared in wild-type COH1, ∆SagCOH1II_RMS, ∆SagCOH1II_RMS__REV_, and BM110 (naturally RMS negative). SagCOH1II was associated with increased transcription of *nanR_GBS_*. Data represent the mean and standard deviation of three independent experiments (one-way ANOVA test with multiple comparisons; ****P < .0001). (**B**) Comparison of *nanR_GBS_* transcription in SagCOH1II_RMS expressing GBS with all other strains. qPCR was performed on all available CC17 GBS (green dots, *n* = 8) and expression of *nanR_GBS_* compared with representative isolates from the five major human-disease-associated serotypes (serotype 1a, grey dots, *n* = 3; serotype II, blue dots, *n* = 2; serotype IV, yellow dots, *n* = 1; and serotype V, orange dots, *n* = 1). Transcription of *nanR_GBS_* is enhanced in CC17 strains expressing SagCOH1II. Data represent the mean value for each group (unpaired *t*-test; ****P < .001). (**C**) Quantitative RT-PCR was used to validate differential expression of *nanR_GBS_* mediated by SagCOH1II. *nanR_GBS_* transcription at OD_600_ 0.9 was compared in WT COH1, ∆SagCOH1II_RMS, ∆SagCOH1II_RMS__REV_, and BM110 (RMS negative). SagCOH1II was associated with increased transcription of *nanR_GBS_*. Data represent the mean and standard deviation of three independent experiments (one-way ANOVA test with multiple comparisons; ***P < .001). (**D**) Schematic representation of the SagCOH1II target methylation site at in the *nanR_GBS_* promoter (red star). ‘Mutated sequence’ depicts the SNP (GGACC-GGAAC) introduced to scramble the SagCOH1II methylation target within the COH1 *nanR_GBS_* promoter, generating strain nanR_GBS__prom_C:A_. (**E**) Comparison of *nanR_GBS_* transcription in WT COH1 with isogenic mutants ∆SagCOH1II_RMS and nanR_GBS__prom_C:A_. Mutation of SagCOH1II target site results in reduction of *nanR_GBS_* transcription. Data represent the mean and standard deviation of three independent experiments (one-way ANOVA test with multiple comparisons; ***P < .001; **P < .01). (**F**) Table depicting the genes with the SagCOH1II target site in the −10 to −35 region within the COH1 genome. (**G**) AlphaFold was used to generate a predicted structure of NanR_GBS_. NanR_GBS_ (green) shares structural similarity with an uncharacterized *Streptococcus pneumoniae* protein [Spr1518 (grey); sequence identity: 49.6%; E-value: 5.53e−26] and the crystal structure of the sialic acid utilization regulator NanR from *Vibrio vulnificus* [Protein data bank (PDB) accession: 4IVN; sequence identity: 20.4%; E-value: 1.53e−13]. Schematic representation of the location of the *nanR* gene in the sialic acid utilization (*nan*) operon; purple star denotes NanR_GBS_ regulatory binding site (TCTGAAACTACTTTCACG) [60]. (**H**) Comparison of COH1, ∆SagCOH1II_RMS, and ∆nanR growth in THB ± sialic acid (2 mM). Growth was comparable amongst all strains in THB, however supplementation with sialic acid significantly enhanced growth of COH1 compared with both mutants. ∆SagCOH1II_RMS showed a more subtle and significantly reduced response to sialic acid availability. Data represent the mean and standard deviation of three independent experiments (two-way ANOVA with multiple comparisons; ***P < .001, **P < .01, *P < .05, ns = *P* > .05).

Deletion of SagCOH1II resulted in a reduction in transcription of a single additional gene, GBSCOH1_0041, at both logarithmic and stationary growth (Fig. 5A and B). These results were validated by RT-PCR including strains COH1∆SagCOH1II_RMS__REV_ and BM110 as controls. Importantly, BM110, a CC17 clinical isolate that is naturally deficient in RMS, exhibited GBSCOH1_0041 expression levels comparable with the SagCOH1II_RMS deletion mutant (Fig. 6A). This led us to hypothesize that increased transcription of GBSCOH1_0041 was a hallmark of the CC17 clone. Comparison of GBSCOH1_0041 transcription across all neonatal disease-associated lineages demonstrated that the presence of SagCOH1II was associated with enhanced expression, and unique to the CC17 strains (Fig. 6B). Differential expression of GBSCOH1_0041 was also confirmed at stationary growth (Fig. 6C).

We identified a SagCOH1II target motif in the GBSCOH1_0041 promoter region, 12 bp upstream of the start codon (Fig. 6D). Nanopore sequence data generated for COH1 and ∆SagCOH1II_RMS were interrogated for DNA modifications using Nanomotif [50]. Methylation of this site was detected in DNA purified from COH1 and not ∆SagCOH1II_RMS confirming that methylation is dependent on SagCOH1II_RMS. We thus went on to generate a GBSCOH1_0041 promoter mutant in strain COH1, in which the methylation target sequence 5′-GGACC-3′ is mutated to 5′-GGAAC-3′, and can thus no longer be modified (Fig. 6D). Transcription of GBSCOH1_0041 was then quantified by qRT-PCR and compared with the wild-type parental strain and SagCOH1II deletion mutant. Mutation of the methylation target site was sufficient to reduce transcription to levels equivalent to those observed for the RMS deletion mutant, indicating that methylation by SagCOH1II directly regulates transcription of GBSCOH1_0041 (Fig. 6E).

Since we observed that only a single gene was epigenetically regulated by SagCOH1II, we quantified the number and location of SagCOH1II target sequences (5′-GGNCC-3′) within the COH1 genome. Of the 1429 target sequences identified only 46 were located within intergenic regions, and of these only 3 were located within the −10 to −35 region upstream of the associated gene. The target site upstream of the GBSCOH1_0041 was the closest to the transcriptional start site (bases −12 to −8) (Fig. 6F) and thus is consistent with the hypothesis that GBSCOH1_0041 was a key target for epigenetic regulation by SagCOH1II.

### GBSCOH1_0041 encodes the transcriptional regulator NanR

To explore the potential function of the GBSCOH1_0041 gene product, AlphaFold2 was used to generate a predicted structure of the translated protein (Fig. 6G). The highest correlation was found with a predicted structure of the NanR protein expressed by *S. pneumoniae*. The second highest correlation was with a NanR homologue expressed by the Gram-negative pathogen *V. vulnificus* complexed with its regulatory ligand N-acetylmannosamine-6-phosphate (ManNAc-6P) (Fig. 6G) [59]. These data strongly suggest that GBSCOH1_0041 is the GBS homologue of the highly conserved regulatory protein NanR [60, 61].

NanR proteins belong to the GntR family of transcriptional regulators, and control genes essential for sialic acid catabolism. These genes are often found in clusters within the genome, collectively forming the *nan* operon that is pervasive across Gram-positive and negative species [59, 62–64]. NanR has been well characterized in the Gram-positive pathogen *S. pneumoniae*, where it has been shown to positively regulate expression of the sialic acid catabolism genes [60]. Given the phylogenetic proximity of *S. pneumoniae* with GBS [65], we hypothesized that GBSCOH1_0041 would mimic the behaviour of the *S. pneumoniae* homologue, and named the gene *nanR_GBS_*. We went on to show that the gene is ubiquitous amongst all GBS lineages (Supplementary Fig. S2). Further analysis of the COH1 genome revealed that *nanR_GBS_* is located 3′ to the previously identified GBS *nan* operon, upstream of which we identified a putative NanR binding site (Fig. 6G) [61]. These *in silico* data led us to hypothesize that NanR_GBS_ regulates sialic acid catabolism in GBS, and increased expression mediated by SagCOH1II is associated with the success of the CC17 clade.

**Figure 7.**
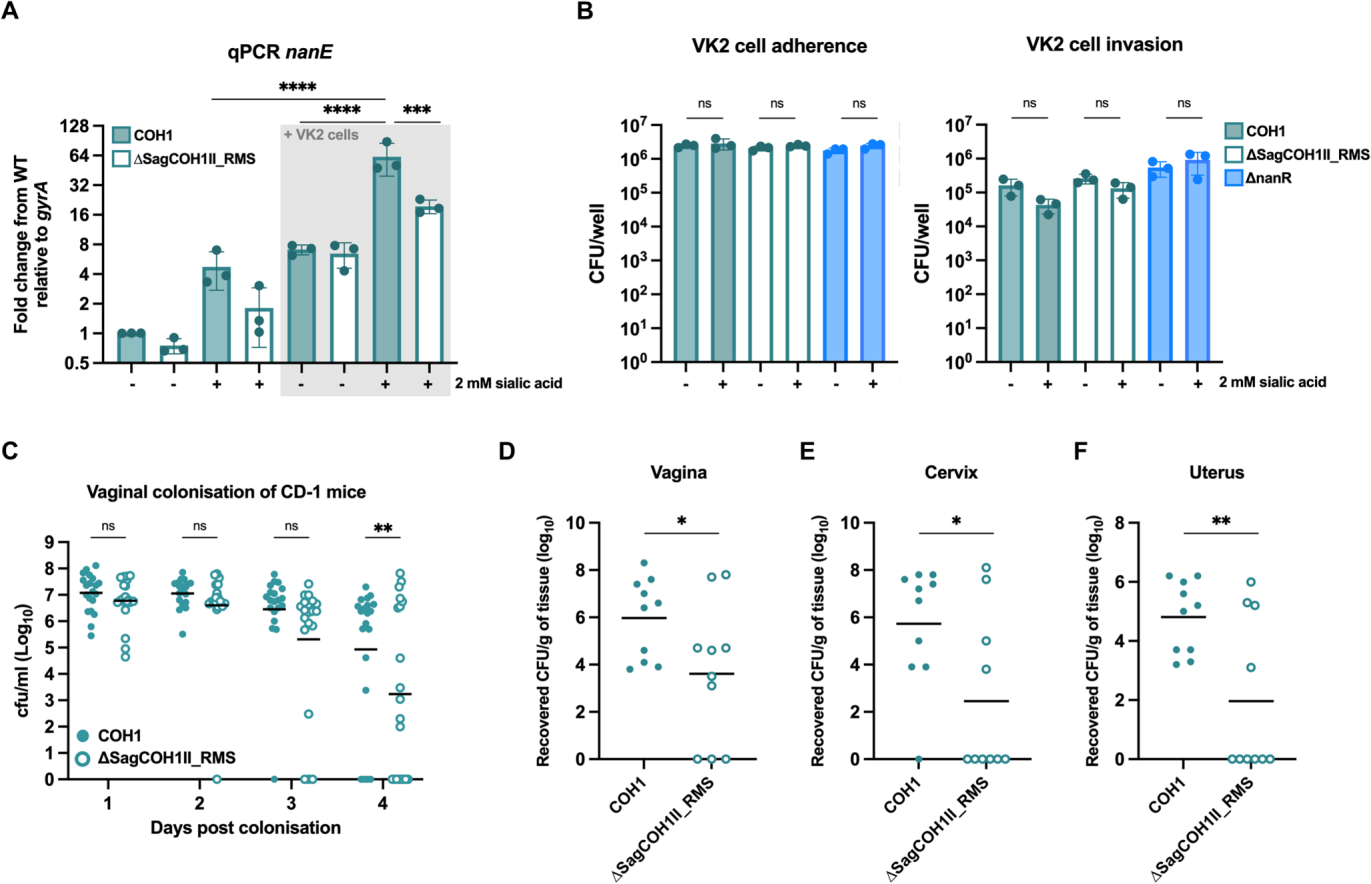
SagCOH1II contributes to the success of CC17 GBS *in vivo*. (**A**) SagCOH1II_RMS is required for effective induction of *nan* operon gene expression in KSFM. Quantitative RT-PCR of the gene *nanE* was used to assess the impact of SagCOH1II_RMS on the induction of gene expression from the *nan* operon in CC17 GBS following exposure to VK2 cells and sialic acid (2 mM). Significant induction of *nanE* expression was seen following COH1 culture in media alone or VK2 cell culture following supplementation with sialic acid. *nanE* expression was significantly greater for COH1 compared with ∆SagCOH1II_RMS following culture with VK2 cells + sialic acid. Data represent mean and standard deviation of three independent experiments (one-way ANOVA test with multiple comparisons; ****P < .0001; ***P < .001; ns = *P* > .05 for COH1 in KSFM versus COH1 in KSFM + SA, ∆SagCOH1II_RMS in KSFM, ∆SagCOH1II_RMS in KSFM + SA, COH1 + VK2; ∆SagCOH1II_RMS in KSFM versus ∆SagCOH1II_RMS in KSFM + SA, ∆SagCOH1II_RMS + VK2; and ∆SagCOH1II_RMS in KSFM + SA versus ∆SagCOH1II_RMS + VK2 + SA). (**B**) Adhesion and invasion of COH1, SagCOH1II_RMS and ∆nanR to VK2 cells was compared by quantitative culture. Data represent mean ± SD of three experimental replicates (*t*-test ns = *P* > .05). (C–F) Mice were inoculated directly into their vaginal tract with 1 × 10^7^ CFU of GBS strains COH1 (wild type, filled symbols) or isogenic mutant COH1∆SagCOH1II_RMS (empty symbols). Recovered CFU counts from daily lavage are shown (**C**). Lavage data are shown from two independent experiments (*n* = 10 mice/group, per experiment) (two-way ANOVA with uncorrected Fisher’s LSD test; **P < .01; ns = *P *> .05). Four days post-inoculation, the vagina (**D**), cervix (**E**), and uterus (**F**) were harvested, homogenized, and GBS CFU enumerated. Tissue data are shown from one independent experiment. Line represents the mean value for each group (*n* = 10 mice per group, over multiple cages). (Unpaired *t*-test with Welch’s correction; *P < .05, **P < .01).

To test this hypothesis, and ascertain whether SagCOH1II impacts the sensitivity of CC17 GBS to sialic acid, bacterial growth assays were performed comparing COH1 with ∆SagCOH1II_RMS. A *nanR* deletion mutant, COH1∆nanR, was generated to provide a non-responsive control strain. While no difference in growth was observed for COH1 compared with ∆SagCOH1II_RMS or ∆nanR in THB alone, following supplementation of media with exogenous sialic acid, COH1 exhibited a significant increase in growth compared to all other strains. An intermediate phenotype was observed for ∆SagCOH1II_RMS (Fig. 7A). These data strongly indicate that SagCOH1II activity increases the sensitivity of CC17 GBS to sialic acid, potentially contributing to the enhanced fitness of this lineage *in vivo*.

### SagCOH1II promotes GBS murine colonization and ascension to the uterus

Mucosal surfaces, including those lining human gastrointestinal and urogenital tracts, are rich in glycoproteins that are heavily decorated with sialic acid residues. We thus hypothesized that the clade-specific enhanced expression of *nanR_GBS_* in CC17 GBS might provide enhanced sensitivity to exogenous sialic acid, and a fitness advantage in this niche.

We initially sought to discern the importance of SagCOH1II activity during colonization of the vaginal epithelium by performing infections of human vaginal epithelial cells (the VK2 cell line [66]), which have already been shown to support enhanced adherence of CC17 GBS [67]. Importantly, in GBS the neuraminidase (*nanA*) gene encoded by the *nan* operon has been inactivated [68], however the rest of the operon is still functional, facilitating uptake and utilization of sialic acid as a carbon source [61]. We thus hypothesized that the *nan* operon in GBS enables scavenging of free sialic acid at mucosal surfaces such as the vaginal tract, where it has been released by neuraminidases expressed by other members of the microbiome. The operon would thus have the potential to be functionally active only where polymicrobial communities are present, but not at sterile sites in the body or 2D cell culture [69]. Experimental infection of VK2 cells was thus performed in the presence and absence of sialic acid, to better model physiologically relevant conditions.

We first set out to confirm whether the GBS *nan* operon is switched on during infection of VK2 cells, and subsequently to ascertain whether sialic acid is involved in transcriptional activation of this operon in GBS. Expression of *nanE*, the first gene in the *nan* operon (Fig. 6G), was quantified for COH1 and ∆SagCOH1II_RMS in cell culture media (KSFM) alone and during VK2 cell infection in the presence and absence of 2 mM sialic acid (Fig. 7A). Expression of *nanE* was significantly enhanced in wild-type strain COH1 following infection of VK2 cells in the presence of sialic acid when compared with transcription following either culture in KSFM + sialic acid or infection of VK2 cells alone. No difference was observed for the SagCOH1II deletion mutant in the same conditions (Fig. 7A). Importantly, expression of *nanE* was significantly higher in COH1 than ∆SagCOH1II_RMS following infection of VK2 cells in the presence of sialic acid, validating the growth phenotype reported here (Fig. 6H) and indicating that in physiologically relevant conditions, SagCOH1II enhances the sensitivity of CC17 GBS to sialic acid.

Despite the clear differences in expression of the *nan* operon between COH1 and ∆SagCOH1II_RMS during VK2 cell infection with exogenous sialic acid, no difference was observed between these strains in either adherence to or invasion of VK2 cells (Fig. 7B). Interestingly, similar results were obtained for COH1∆nanR, where addition of exogenous sialic acid also had no effect on VK2 cell infection by either COH1 or ∆SagCOH1II_RMS (Fig. 7B). Given the clear effect of sialic acid on *nanE* transcription in COH1, these data suggest that this assay was not sufficiently sensitive to distinguish differences in sialic acid response or associated GBS virulence, indicating a more complex and physiologically relevant system was required.

We thus sought to discern the importance of RMS activity at mucosal surfaces *in vivo*, where free sialic acid released by members of the microbiome would be available. As the female reproductive tract is an important reservoir for GBS, and is associated with sialic acid-degrading species within the microbiome [70, 71], we compared the ability of COH1 and the isogenic SagCOH1II deletion mutant to colonize the vaginal tract using a well-established murine model [72, 73]. Following intra-vaginal inoculation, bacterial colonization was assessed by daily lavage of the vaginal lumen. We observed that both the wild-type and SagCOH1II deletion mutant strains were able to colonize the lumen initially, but over time we recovered less CFU from mice colonized with the SagCOH1II deletion mutant (Fig. 7C). Overall, there was a significant reduction in bacterial burden for mice infected with the SagCOH1II deletion mutant compared with wild-type COH1, suggesting that methylation mediated by SagCOH1II may promote CC17 GBS vaginal colonization and persistence.

The attenuation in virulence following deletion of SagCOH1II was also observed in vaginal (Fig. 7D), cervical (Fig. 7E), and uterine (Fig. 7F) tissues collected on day 4 post-colonization. These data suggest that SagCOH1II not only enhances CC17 fitness in the vaginal lumen, but also at mucosal surfaces, and subsequent ascension to the uterus. Combined, these data support a role for SagCOH1II in driving *in vivo* fitness of CC17 GBS, potentially contributing to the hypervirulence associated with this lineage.

## Discussion

Highly virulent lineages of bacterial pathogens can evolve by clonal expansion, a process associated with genetic uniformity following selection for a highly fit genotype within a lineage [74–76]. Defining the bacterial pressures that drive this process is of key importance in understanding the emergence of pathogenic lineages, in particular those that pose an epidemic threat. The CC17 lineage of GBS provides a classic example of clonality [77], and thus understanding the underlying genetic pressures that underpin its emergence are of wide relevance. The contribution of RMS activity to clonal expansion of bacterial pathogens remains poorly characterized, but is potentially critical. By combining bioinformatic analysis of DNA recombination within the GBS genome with experimental transformation efficiency analyses, we have been able to demonstrate a direct contribution of RMS activity to the clonality of the CC17 lineage, and suggest that this association may be broadly conserved among diverse bacterial pathogens.

While it is now relatively easy to identify RMS genes in bacterial genomes *in silico* using freely available databases [39], these data are insufficient to attribute activity or evolutionary impact to a novel operon. An inter-disciplinary approach, combining bioinformatics and population genomics with bacterial genetics is thus essential to robustly interrogate identified operons, and accurately assess their contribution to bacterial evolution and physiology. Here, we have used Nanopore sequencing to establish the methylation profile of CC17 gDNA, and define the target motif for each RMS associated with this lineage.

The mere presence of RMS genes or their associated recognition motifs within a bacterial genome does not necessarily indicate functional enzymatic activity [78, 79]. Moreover, not all detected DNA methylation events have a measurable impact on DNA structure or gene regulation, emphasizing that the presence of methylation modifications alone does not imply biological function. The efficiency of restriction enzymes themselves may also be insufficient to substantially influence DNA uptake, particularly if expression levels or enzyme activity are low. Such efficiency is further shaped by the expression dynamics of individual genes within the RMS, which can vary considerably across environmental conditions and strains [80, 81]. Consequently, any predicted restriction-mediated inhibition of DNA uptake must be supported by experimental validation to confirm physiological relevance. In support of this, only one of the identified CC17-associated RMS (SagCOH1II) was able to elicit an effect on genetic flux or transcription, highlighting the importance of experimentally validating RMS activity *in vivo*. Highly active restriction endonucleases can significantly complicate genetic manipulation and subsequent study of often highly virulent and clinically important lineages. Generation of passaging strains utilized to protectively methylate engineered DNA can alleviate this issue [82], and a strain to overcome restriction mediated by SagCOH1II is under development currently.

RMS activity can impact bacterial evolution not only by restricting gene flow, but also epigenetic modulation of gene expression [21, 28, 29, 31, 32], thereby driving not only the emergence, but also the maintenance of phenotypically distinct lineages [19, 22]. RMS with combined restriction and methyltransferase activity can also act as phage defence operons [24], and while beyond the scope of this study, our data support a role for SagCOH1II in phage defence, with the potential to further impact the fitness and evolution of CC17 GBS. RMS are frequently acquired by horizontal gene transfer [83], and while acquisition can be toxic to the bacterial host, careful regulation of RMS gene expression can result in successful incorporation into the bacterial genome [84], as demonstrated in this report. Acquisition of novel RMS by horizontal gene transfer can provide a fitness advantage through modulation of gene expression [32, 85], as we have also shown for SagCOH1II (Fig. 7C–F).

The exact mechanisms by which methylation as a DNA modification alter transcription remain incompletely understood, however it is postulated to modulate the interaction of DNA with important DNA binding proteins including RNA polymerase and specific transcription factors [86]. While research into these mechanisms is beyond the scope of this manuscript, RNAseq analysis enabled us to experimentally define an epigenetic role for RMS activity in CC17 GBS, consistent with this hypothesis. Deletion of SagCOH1II and subsequent loss of DNA methylation specifically reduced transcription of the sialic acid utilization regulator *nanR*_GBS_, a feature we were able to show is a CC17-specific phenomenon and defining characteristic of the lineage. The location of the methylation target site (−10 bp from ATG) strongly implicates altered DNA binding efficiency of regulatory proteins as the mechanism of increased gene expression amongst CC17 strains, a potentially common feature across biology.

Using genetic data like these is critical to better understand the phenotypic fitness benefits associated with emergent lineages. Ultimately this may facilitate the development of new and efficient infection control strategies. GBSCOH1_0041 encodes a homologue of the sialic acid utilization regulator NanR [60], and the increased expression of this gene by the CC17 lineage was associated with increased growth and transcription from the *nan* operon during exposure to exogenous sialic acid (Figs 6H and 7A), providing a clear rationale for enhanced survival of CC17 GBS at sites where sialic acid is readily available as a carbon source. Sialic acid decorates surface glycans expressed at mucosal surfaces [71], and similar to reports for the pathogen *S. pneumoniae* [60], we have shown SagCOH1II activity promotes survival at mucosal surfaces and enhances subsequent ascension to normally sterile sites (Fig. 7D–F).

The importance of sialic acid as a carbon source for bacteria during colonization and invasion of mucosal surfaces is well-documented [60, 61, 64]. While not studied in detail, *nanR*_GBS_ has previously been implicated in GBS colonization and subsequent ascending infection of the vaginal tract in published genome-wide screens [87, 88], and our findings provide evidence of the functional relevance of this gene. The GBS neuraminidase gene (*nanA*) has been inactivated, thus GBS depend on the activity of resident microbiome species for the release of sialic acid from mucosal surfaces. *nanR_GBS_* would thus not be expected to contribute to fitness following infection of normally sterile sites lacking free sialic acid, in line with observations for human blood [89], plasma [89], and amniotic fluid [90] in similar studies. Enhanced expression of the *nan* operon would require free sialic acid provided by exogenous supplementation, as demonstrated in our study, or due to the presence of neuraminidase-producing microbiome species such as *Akkermansia muciniphila*, as shown for COH1 during VK2 cell infection [57, 91]. Additional published data have shown that the *nanR*_GBS_ gene is uniquely under positive selection in CC17 strains [92], indicating a distinct evolutionary advantage attributed to the gene product in this lineage. We thus predict that the enhanced clade-specific expression of *nanR_GBS_* by CC17 strains provides a fitness advantage at polymicrobial mucosal surfaces, possibly by improving growth and subsequent tissue invasion in these hostile environments.

RMS have long been recognized as key defence mechanisms against invading foreign DNA including predation by phage, and emerging evidence suggests that their roles extend well beyond host protection [23]. In this study, we demonstrate that a previously uncharacterized RMS conserved within the hypervirulent CC17 lineage of GBS exerts dual regulatory functions, modulating genetic flux and controlling the transcriptional landscape of metabolic pathways potentially driving bacterial survival at and ascending infection from polymicrobial mucosal surfaces. In conclusion, our findings underscore a broader paradigm in which RMS serve not only as barriers to genetic invasion but also as lineage-specific regulators of gene expression and carbon source utilization. This dual functionality may be a key factor in the ecological success and pathogenic potential of certain bacterial strains, particularly at mucosal surfaces where environmental cues and host interactions exert strong selective forces.

## Supplementary Material

gkag683_Supplemental_Files

## Data Availability

The sequencing data generated in this study have been deposited in the European Nucleotide Archive (ENA, https://www.ebi.ac.uk/ena/browser/) under accession number PRJEB94460. The data are publicly available upon publication.

## References

[1] Le Doare K, Heath PT. An overview of global GBS epidemiology. Vaccine. 2013;31:D7–12. 23973349 10.1016/j.vaccine.2013.01.009

[2] Seale AC, Bianchi-Jassir F, Russell NJ et al. Estimates of the burden of group B streptococcal disease worldwide for pregnant women, stillbirths, and children. Clin Infect Dis. 2017;65:S200–19. 10.1093/cid/cix664 29117332 PMC5849940

[3] Almeida A, Rosinski-Chupin I, Plainvert C et al. Parallel evolution of group B *Streptococcus* hypervirulent clonal complex 17 unveils new pathoadaptive mutations. mSystems. 2017;2:e00074–17. 10.1128/mSystems.00074-17 28904998 PMC5585690

[4] Deshayes de Cambronne R, Fouet A, Picart A et al. CC17 group B *Streptococcus* exploits integrins for neonatal meningitis development. J Clin Invest. 2021;131:e136737. 33465054 10.1172/JCI136737 PMC7919713

[5] Jamrozy D, Bijlsma MW, de Goffau MC et al. Increasing incidence of group B *Streptococcus* neonatal infections in the Netherlands is associated with clonal expansion of CC17 and CC23. Sci Rep. 2020;10:9539. 10.1038/s41598-020-66214-3 32533007 PMC7293262

[6] Six A, Bellais S, Bouaboud A et al. Srr2, a multifaceted adhesin expressed by ST-17 hypervirulent group B *Streptococcus* involved in binding to both fibrinogen and plasminogen. Mol Microbiol. 2015;97:1209–22. 10.1111/mmi.13097 26094503

[7] Tazi A, Disson O, Bellais S et al. The surface protein HvgA mediates group B *Streptococcus* hypervirulence and meningeal tropism in neonates. J Exp Med. 2010;207:2313–22. 10.1084/jem.20092594 20956545 PMC2964583

[8] Gori A, Harrison OB, Mlia E et al. Pan-GWAS of *Streptococcus agalactiae* highlights lineage-specific genes associated with virulence and niche adaptation. mBio. 2020;11:e00728–20. 10.1128/mBio.00728-20 32518186 PMC7373188

[9] Da Cunha V, Davies MR, Douarre PE et al. *Streptococcus agalactiae* clones infecting humans were selected and fixed through the extensive use of tetracycline. Nat Commun. 2014;5:4544. 25088811 10.1038/ncomms5544 PMC4538795

[10] Crestani C, Forde TL, Bell J et al. Genomic and functional determinants of host spectrum in group B *Streptococcus*. PLoS Pathog. 2024;20:e1012400. 10.1371/journal.ppat.1012400 39133742 PMC11341095

[11] Torrance EL, Burton C, Diop A et al. Evolution of homologous recombination rates across bacteria. Proc Natl Acad Sci USA. 2024;121:e2316302121. 10.1073/pnas.2316302121 38657048 PMC11067023

[12] Sanchez-Romero MA, Cota I, Casadesus J. DNA methylation in bacteria: from the methyl group to the methylome. Curr Opin Microbiol. 2015;25:9–16. 10.1016/j.mib.2015.03.004 25818841

[13] Kennedy NW, Comstock LE. Mechanisms of bacterial immunity, protection, and survival during interbacterial warfare. Cell Host Microbe. 2024;32:794–803. 10.1016/j.chom.2024.05.006 38870897 PMC11216714

[14] Barrangou R, Fremaux C, Deveau H et al. CRISPR provides acquired resistance against viruses in prokaryotes. Science. 2007;315:1709–12. 10.1126/science.1138140 17379808

[15] Picton DM, Luyten YA, Morgan RD et al. The phage defence island of a multidrug resistant plasmid uses both BREX and type IV restriction for complementary protection from viruses. Nucleic Acids Res. 2021;49:11257–73. 10.1093/nar/gkab906 34657954 PMC8565348

[16] Pastuszka A, Mazzuoli MV, Crestani C et al. The virulence regulator CovR boosts CRISPR–Cas9 immunity in Group B *Streptococcus*. Nat Commun. 2025;16:5678. 10.1038/s41467-025-60871-6 40593653 PMC12216829

[17] Budroni S, Siena E, Dunning Hotopp JC et al. *Neisseria meningitidis* is structured in clades associated with restriction modification systems that modulate homologous recombination. Proc Natl Acad Sci USA. 2011;108:4494–9. 10.1073/pnas.1019751108 21368196 PMC3060241

[18] Banerjee S, Chowdhury R. An orphan DNA (cytosine-5-)-methyltransferase in *Vibrio cholerae*. Microbiology. 2006;152:1055–62. 10.1099/mic.0.28624-0 16549669

[19] Oliveira PH, Touchon M, Rocha EP. Regulation of genetic flux between bacteria by restriction-modification systems. Proc Natl Acad Sci USA. 2016;113:5658–63. 10.1073/pnas.1603257113 27140615 PMC4878467

[20] Johnston CD, Cotton SL, Rittling SR et al. Systematic evasion of the restriction-modification barrier in bacteria. Proc Natl Acad Sci USA. 2019;116:11454–9. 10.1073/pnas.1820256116 31097593 PMC6561282

[21] Li J, Li JW, Feng Z et al. Epigenetic switch driven by DNA inversions dictates phase variation in *Streptococcus pneumoniae*. PLoS Pathog. 2016;12:e1005762. 10.1371/journal.ppat.1005762 27427949 PMC4948785

[22] Blow MJ, Clark TA, Daum CG et al. The Epigenomic landscape of prokaryotes. PLoS Genet. 2016;12:e1005854. 10.1371/journal.pgen.1005854 26870957 PMC4752239

[23] Vasu K, Nagaraja V. Diverse functions of restriction-modification systems in addition to cellular defense. Microbiol Mol Biol Rev. 2013;77:53–72. 10.1128/MMBR.00044-12 23471617 PMC3591985

[24] Anton BP, Blumenthal R, Eaglesham JB et al. Biology of host-dependent restriction-modification in prokaryotes. EcoSal Plus. 2025;13:eesp00142022. 10.1128/ecosalplus.esp-0014-2022 40856689 PMC12707154

[25] Roberts GA, Houston PJ, White JH et al. Impact of target site distribution for Type I restriction enzymes on the evolution of methicillin-resistant *Staphylococcus aureus* (MRSA) populations. Nucleic Acids Res. 2013;41:7472–84. 10.1093/nar/gkt535 23771140 PMC3753647

[26] Lin LF, Posfai J, Roberts RJ et al. Comparative genomics of the restriction-modification systems in *Helicobacter pylori*. Proc Natl Acad Sci USA. 2001;98:2740–5. 10.1073/pnas.051612298 11226310 PMC30209

[27] Nye TM, Jacob KM, Holley EK et al. DNA methylation from a Type I restriction modification system influences gene expression and virulence in *Streptococcus pyogenes*. PLoS Pathog. 2019;15:e1007841. 10.1371/journal.ppat.1007841 31206562 PMC6597129

[28] Balbontin R, Rowley G, Pucciarelli MG et al. DNA adenine methylation regulates virulence gene expression in *Salmonella enterica* serovar Typhimurium. J Bacteriol. 2006;188:8160–8. 10.1128/JB.00847-06 16997949 PMC1698197

[29] Chao MC, Zhu S, Kimura S et al. A cytosine methyltransferase modulates the cell envelope stress response in the *Cholera* pathogen [corrected]. PLoS Genet. 2015;11:e1005666. 10.1371/journal.pgen.1005666 26588462 PMC4654547

[30] Heithoff DM, Sinsheimer RL, Low DA et al. An essential role for DNA adenine methylation in bacterial virulence. Science. 1999;284:967–70. 10.1126/science.284.5416.967 10320378

[31] Kumar S, Karmakar BC, Nagarajan D et al. N4-cytosine DNA methylation regulates transcription and pathogenesis in *Helicobacter pylori*. Nucleic Acids Res. 2018;46:3429–45. 10.1093/nar/gky126 29481677 PMC5909468

[32] Roodsant TJ, van der Putten B, Brizuela J et al. The streptococcal phase-variable type I restriction modification system SsuCC20p dictates the methylome of *Streptococcus suis* impacting the transcriptome and virulence in a zebrafish larvae infection model. mBio. 2024;15:e0225923. 10.1128/mbio.02259-23 38063379 PMC10790761

[33] Atack JM, Weinert LA, Tucker AW et al. *Streptococcus suis* contains multiple phase-variable methyltransferases that show a discrete lineage distribution. Nucleic Acids Res. 2018;46:11466–76. 30304532 10.1093/nar/gky913 PMC6265453

[34] Wilson CB, Weaver WM. Comparative susceptibility of group B streptococci and *Staphylococcus aureus* to killing by oxygen metabolites. J Infect Dis. 1985;152:323–9. 10.1093/infdis/152.2.323 2993435

[35] Lancefield RC, McCarty M, Everly WN. Multiple mouse-protective antibodies directed against group B streptococci. Special reference to antibodies effective against protein antigens. J Exp Med. 1975;142:165–79. 10.1084/jem.142.1.165 1097573 PMC2189884

[36] Wessels MR, Paoletti LC, Rodewald AK et al. Stimulation of protective antibodies against type Ia and Ib group B streptococci by a type Ia polysaccharide-tetanus toxoid conjugate vaccine. Infect Immun. 1993;61:4760–6. 10.1128/iai.61.11.4760-4766.1993 8406875 PMC281231

[37] van Sorge NM, Bonsor DA, Deng L et al. Bacterial protein domains with a novel Ig-like fold target human CEACAM receptors. EMBO J. 2021;40:e106103. 33522633 10.15252/embj.2020106103 PMC8013792

[38] Stalhammar-Carlemalm M, Stenberg L, Lindahl G. Protein rib: a novel group B streptococcal cell surface protein that confers protective immunity and is expressed by most strains causing invasive infections. J Exp Med. 1993;177:1593–603. 10.1084/jem.177.6.1593 8496678 PMC2191029

[39] Roberts RJ, Vincze T, Posfai J et al. REBASE: a database for DNA restriction and modification: enzymes, genes and genomes. Nucleic Acids Res. 2023;51:D629–30. 10.1093/nar/gkac975 36318248 PMC9825431

[40] Camacho C, Coulouris G, Avagyan V et al. BLAST+: architecture and applications. BMC Bioinformatics. 2009;10:421. 10.1186/1471-2105-10-421 20003500 PMC2803857

[41] Nguyen LT, Schmidt HA, von Haeseler A et al. IQ-TREE: a fast and effective stochastic algorithm for estimating maximum-likelihood phylogenies. Mol Biol Evol. 2015;32:268–74. 10.1093/molbev/msu300 25371430 PMC4271533

[42] Croucher NJ, Page AJ, Connor TR et al. Rapid phylogenetic analysis of large samples of recombinant bacterial whole genome sequences using Gubbins. Nucleic Acids Res. 2015;43:e15. 10.1093/nar/gku1196 25414349 PMC4330336

[43] Hadfield J, Croucher NJ, Goater RJ et al. Phandango: an interactive viewer for bacterial population genomics. Bioinformatics. 2018;34:292–3. 10.1093/bioinformatics/btx610 29028899 PMC5860215

[44] Alves J, Dry I, White JH et al. Generation of tools for expression and purification of the phage-encoded Type I restriction enzyme inhibitor, Ocr. Microbiology (Reading). 2024;170:001465. 10.1099/mic.0.001465 39042422 PMC11317969

[45] Alves J, Rand JD, Johnston ABE et al. Methylome-dependent transformation of emm1 group A streptococci. mBio. 2023;14:e0079823. 10.1128/mbio.00798-23 37427929 PMC10470502

[46] Lynskey NN, Goulding D, Gierula M et al. RocA truncation underpins hyper-encapsulation, carriage longevity and transmissibility of serotype M18 group A streptococci. PLoS Pathog. 2013;9:e1003842. 10.1371/journal.ppat.1003842 24367267 PMC3868526

[47] Perez-Casal J, Price JA, Maguin E et al. An M protein with a single C repeat prevents phagocytosis of *Streptococcus pyogenes*: use of a temperature-sensitive shuttle vector to deliver homologous sequences to the chromosome of *S. pyogenes*. Mol Microbiol. 1993;8:809–19. 10.1111/j.1365-2958.1993.tb01628.x 8355608

[48] Danecek P, Bonfield JK, Liddle J et al. Twelve years of SAMtools and BCFtools. GigaScience. 2021;10:giab008. 10.1093/gigascience/giab008 33590861 PMC7931819

[49] Kolmogorov M, Yuan J, Lin Y et al. Assembly of long, error-prone reads using repeat graphs. Nat Biotechnol. 2019;37:540–6. 10.1038/s41587-019-0072-8 30936562

[50] Heidelbach S, Dall SM, Bøjer JS et al. Nanomotif: leveraging DNA methylation motifs for genome recovery and host association of plasmids in metagenomes from complex microbial communities. bioRxiv, 10.1101/2024.04.29.591623, 4 January, 2025, preprint; not peer reviewed

[51] Jumper J, Evans R, Pritzel A et al. Highly accurate protein structure prediction with AlphaFold. Nature. 2021;596:583–9. 10.1038/s41586-021-03819-2 34265844 PMC8371605

[52] van Kempen M, Kim SS, Tumescheit C et al. Fast and accurate protein structure search with Foldseek. Nat Biotechnol. 2024;42:243–6. 37156916 10.1038/s41587-023-01773-0 PMC10869269

[53] Brokaw A, Wallen G, Orvis A et al. The serine protease HtrA regulates Group B *Streptococcus* virulence and affects the host response to infection. PLoS Pathog. 2025;21:e1013562. 10.1371/journal.ppat.1013562 41052219 PMC12520345

[54] Vollmuth N, Sauerwein T, Foerstner KU et al. *Streptococcus agalactiae* strain COH1 transcriptome in association with stem cell-derived brain-like endothelial cells. Microbiol Resour Announc. 2024;13:e0045524. 10.1128/mra.00455-24 39526785 PMC11636360

[55] Joyce LR, Kim S, Spencer BL et al. *Streptococcus agalactiae* glycolipids promote virulence by thwarting immune cell clearance. Sci Adv. 2024;10:eadn7848. 10.1126/sciadv.adn7848 38809989 PMC11135403

[56] Doro F, Liberatori S, Rodriguez-Ortega MJ et al. Surfome analysis as a fast track to vaccine discovery: identification of a novel protective antigen for Group B Streptococcus hypervirulent strain COH1. Mol Cell Proteomics. 2009;8:1728–37. 10.1074/mcp.M800486-MCP200 19401597 PMC2709197

[57] Marroquin SM, Cohen S, Neely MN et al. *Akkermansia muciniphila* impacts group B *Streptococcus* vaginal colonization. mBio. 2026;17:e0286825. 10.1128/mbio.02868-25 42041249 PMC13251365

[58] Crestani C, Forde TL, Zadoks RN. Development and application of a prophage integrase typing scheme for Group B *Streptococcus*. Front Microbiol. 2020;11:1993. 10.3389/fmicb.2020.01993 32983017 PMC7487436

[59] Hwang J, Kim BS, Jang SY et al. Structural insights into the regulation of sialic acid catabolism by the *Vibrio vulnificus* transcriptional repressor NanR. Proc Natl Acad Sci USA. 2013;110:E2829–2837. 10.1073/pnas.1302859110 23832782 PMC3725057

[60] Afzal M, Shafeeq S, Ahmed H et al. Sialic acid-mediated gene expression in *Streptococcus pneumoniae* and role of NanR as a transcriptional activator of the nan gene cluster. Appl Environ Microb. 2015;81:3121–31. 10.1128/AEM.00499-15 PMC4393437 25724955

[61] Pezzicoli A, Ruggiero P, Amerighi F et al. Exogenous sialic acid transport contributes to group B streptococcus infection of mucosal surfaces. J Infect Dis. 2012;206:924–31. 10.1093/infdis/jis451 22829646

[62] Horne CR, Venugopal H, Panjikar S et al. Mechanism of NanR gene repression and allosteric induction of bacterial sialic acid metabolism. Nat Commun. 2021;12:1988. 10.1038/s41467-021-22253-6 33790291 PMC8012715

[63] Li J, Evans DR, Freedman JC et al. NanR regulates NanI sialidase expression by *Clostridium perfringens* F4969, a human enteropathogenic strain. Infect Immun. 2017;85:e00241–17. 10.1128/IAI.00241-17 28652312 PMC5563580

[64] Brigham C, Caughlan R, Gallegos R et al. Sialic acid (N-acetyl neuraminic acid) utilization by *Bacteroides fragilis* requires a novel N-acetyl mannosamine epimerase. J Bacteriol. 2009;191:3629–38. 10.1128/JB.00811-08 19304853 PMC2681899

[65] Richards VP, Palmer SR, Pavinski Bitar PD et al. Phylogenomics and the dynamic genome evolution of the genus *Streptococcus*. Genome Biol Evol. 2014;6:741–53. 10.1093/gbe/evu048 24625962 PMC4007547

[66] Fichorova RN, Rheinwald JG, Anderson DJ. Generation of papillomavirus-immortalized cell lines from normal human ectocervical, endocervical, and vaginal epithelium that maintain expression of tissue-specific differentiation proteins. Biol Reprod. 1997;57:847–55. 10.1095/biolreprod57.4.847 9314589

[67] Burcham LR, Spencer BL, Keeler LR et al. Determinants of Group B streptococcal virulence potential amongst vaginal clinical isolates from pregnant women. PLoS One. 2019;14:e0226699. 10.1371/journal.pone.0226699 31851721 PMC6919605

[68] Yamaguchi M, Hirose Y, Nakata M et al. Evolutionary inactivation of a sialidase in group B *Streptococcus*. Sci Rep. 2016;6:28852. 10.1038/srep28852 27352769 PMC4926279

[69] Bell A, Juge N. Mucosal glycan degradation of the host by the gut microbiota. Glycobiology. 2021;31:691–6. 10.1093/glycob/cwaa097 33043970 PMC8252862

[70] Pelayo P, Hussain FA, Werlang CA et al. *Prevotella* are major contributors of sialidases in the human vaginal microbiome. Proc Natl Acad Sci USA. 2024;121:e2400341121. 10.1073/pnas.2400341121 39186657 PMC11388281

[71] Agarwal K, Choudhury B, Robinson LS et al. Resident microbes shape the vaginal epithelial glycan landscape. Sci Transl Med. 2023;15:eabp9599. 10.1126/scitranslmed.abp9599 38019934 PMC11419735

[72] Patras KA, Doran KS. A murine model of Group B *Streptococcus* vaginal colonization. J Vis Exp. 2016; 16:54708. 10.3791/54708 PMC5226234 27911391

[73] Patras KA, Rosler B, Thoman ML et al. Characterization of host immunity during persistent vaginal colonization by Group B *Streptococcus*. Mucosal Immunol. 2015;8:1339–48. 10.1038/mi.2015.23 25850655 PMC4598252

[74] Hullahalli K, Waldor MK. Pathogen clonal expansion underlies multiorgan dissemination and organ-specific outcomes during murine systemic infection. eLife. 2021;10:e70910 10.7554/eLife.70910 34636322 PMC8545400

[75] Keenan JD, Klugman KP, McGee L et al. Evidence for clonal expansion after antibiotic selection pressure: pneumococcal multilocus sequence types before and after mass azithromycin treatments. J Infect Dis. 2015;211:988–94. 10.1093/infdis/jiu552 25293366 PMC4416126

[76] McVicker G, Prajsnar TK, Williams A et al. Clonal expansion during *Staphylococcus aureus* infection dynamics reveals the effect of antibiotic intervention. PLoS Pathog. 2014;10:e1003959. 10.1371/journal.ppat.1003959 24586163 PMC3937288

[77] Chaguza C, Jamrozy D, Bijlsma MW et al. Population genomics of Group B *Streptococcus* reveals the genetics of neonatal disease onset and meningeal invasion. Nat Commun. 2022;13:4215. 10.1038/s41467-022-31858-4 35864107 PMC9304382

[78] Chen X, Li H, Lin Q et al. Structure-based design of a dual-warhead covalent inhibitor of FGFR4. Commun Chem. 2022;5:36. 10.1038/s42004-022-00657-9 36697897 PMC9814781

[79] Mehershahi KS, Chen SL. DNA methylation by three Type I restriction modification systems of *Escherichia coli* does not influence gene regulation of the host bacterium. Nucleic Acids Res. 2021;49:7375–88. 10.1093/nar/gkab530 34181709 PMC8287963

[80] Morozova N, Sabantsev A, Bogdanova E et al. Temporal dynamics of methyltransferase and restriction endonuclease accumulation in individual cells after introducing a restriction-modification system. Nucleic Acids Res. 2016;44:790–800. 10.1093/nar/gkv1490 26687717 PMC4737168

[81] Mruk I, Kobayashi I. To be or not to be: regulation of restriction-modification systems and other toxin–antitoxin systems. Nucleic Acids Res. 2014;42:70–86. 10.1093/nar/gkt711 23945938 PMC3874152

[82] Monk IR, Tree JJ, Howden BP et al. Complete bypass of restriction systems for major *Staphylococcus aureus* lineages. mBio. 2015;6:e00308–00315. 10.1128/mBio.00308-15 26015493 PMC4447248

[83] Ma J, Jiang X, Bi H et al. Horizontal acquisition of the Type I restriction-modification system enhances bacterial pathogenicity by mediating methylation of transcription factor-encoding genes. Nucleic Acids Res. 2025;53:gkaf659. 10.1093/nar/gkaf659 40650971 PMC12255300

[84] Negri A, Werbowy O, Wons E et al. Regulator-dependent temporal dynamics of a restriction-modification system’s gene expression upon entering new host cells: single-cell and population studies. Nucleic Acids Res. 2021;49:3826–40. 10.1093/nar/gkab183 33744971 PMC8053105

[85] Doberenz S, Eckweiler D, Reichert O et al. Identification of a *Pseudomonas aeruginosa* PAO1 DNA methyltransferase, its targets, and physiological roles. mBio. 2017;8:e02312–16. 10.1128/mBio.02312-16 28223461 PMC5358918

[86] Low DA, Casadesus J. Clocks and switches: bacterial gene regulation by DNA adenine methylation. Curr Opin Microbiol. 2008;11:106–12. 10.1016/j.mib.2008.02.012 18396448

[87] Manzer HS, Brunetti T, Doran KS. Identification of a DNA-cytosine methyltransferase that impacts global transcription to promote group B streptococcal vaginal colonization. mBio. 2023;14:e0230623. 10.1128/mbio.02306-23 37905908 PMC10746215

[88] Burcham LR, Akbari MS, Alhajjar N et al. Genomic analyses identify manganese homeostasis as a driver of Group B streptococcal vaginal colonization. mBio. 2022;13:e0098522. 10.1128/mbio.00985-22 35658538 PMC9239048

[89] Zhu L, Yerramilli P, Pruitt L et al. Genome-wide assessment of *Streptococcus agalactiae* genes required for survival in human whole blood and plasma. Infect Immun. 2020;88:e00357–20. 10.1128/IAI.00357-20 32747604 PMC7504961

[90] Dammann AN, Chamby AB, Catomeris AJ et al. Genome-wide fitness analysis of group B *Streptococcus* in human amniotic fluid reveals a transcription factor that controls multiple virulence traits. PLoS Pathog. 2021;17:e1009116. 10.1371/journal.ppat.1009116 33684178 PMC7971860

[91] Shuoker B, Pichler MJ, Jin C et al. Sialidases and fucosidases of Akkermansia muciniphila are crucial for growth on mucin and nutrient sharing with mucus-associated gut bacteria. Nat Commun. 2023;14:1833. 10.1038/s41467-023-37533-6 37005422 PMC10067855

[92] Teatero S, Ramoutar E, McGeer A et al. Clonal complex 17 Group B *Streptococcus* strains causing invasive disease in neonates and adults originate from the same genetic pool. Sci Rep. 2016;6:20047. 10.1038/srep20047 26843175 PMC4740736

