# Supplementary material for: Acquisition of a novel restriction modification system regulates genetic flux and gene expression in the hypervirulent and globally disseminated CC17 lineage of group B Streptococcus
Source: Nucleic Acids Res. 2026 Jul 8;54(13):gkag683. doi: 10.1093/nar/gkag683 (PMC13343189; doi:10.1093/nar/gkag683)
Supplement: gkag683_Supplemental_Files [file gkag683_supplemental_files.zip › Supplementary data.pdf]

**Supplementary data**:

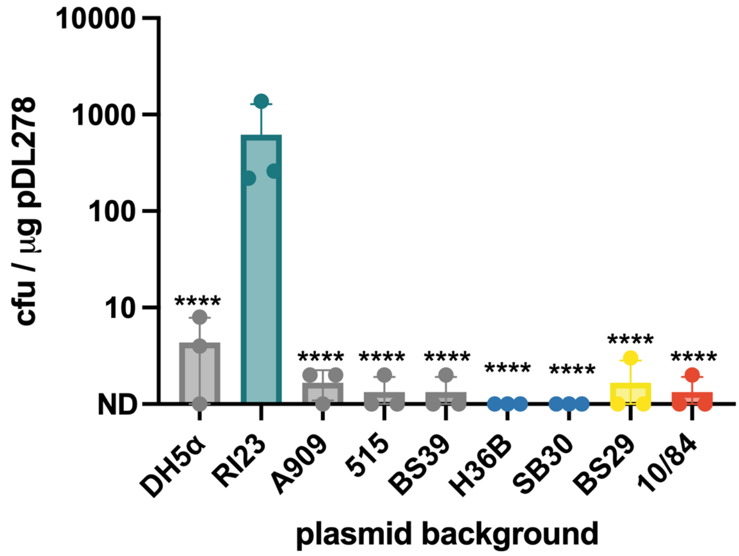


**Supplementary Figure 1: Transformation of CC17 strains is enhanced using self-methylated plasmid DNA**. Comparison of transformation efficiency of CC17 strain RI23 with plasmid pDL278 purified from DH5α- *E. coli* (black bar), Ri23 (green bar) and with representative isolates from the 5 major human-disease associated serotypes (serotype 1a (grey bars, n=3); serotype II (blue bars, n=2); serotype IV (yellow bars, n=1; serotype V (orange bars, n=1). Transformation efficiency of RI23 was enhanced with self-methylated plasmid. Data represent the mean and standard deviation of four independent experiments (one-way ANOVA test with multiple comparisons between plasmid purified from each clinical isolate vs RI23 performed on log-transformed data; ****$p < 0.0001$).

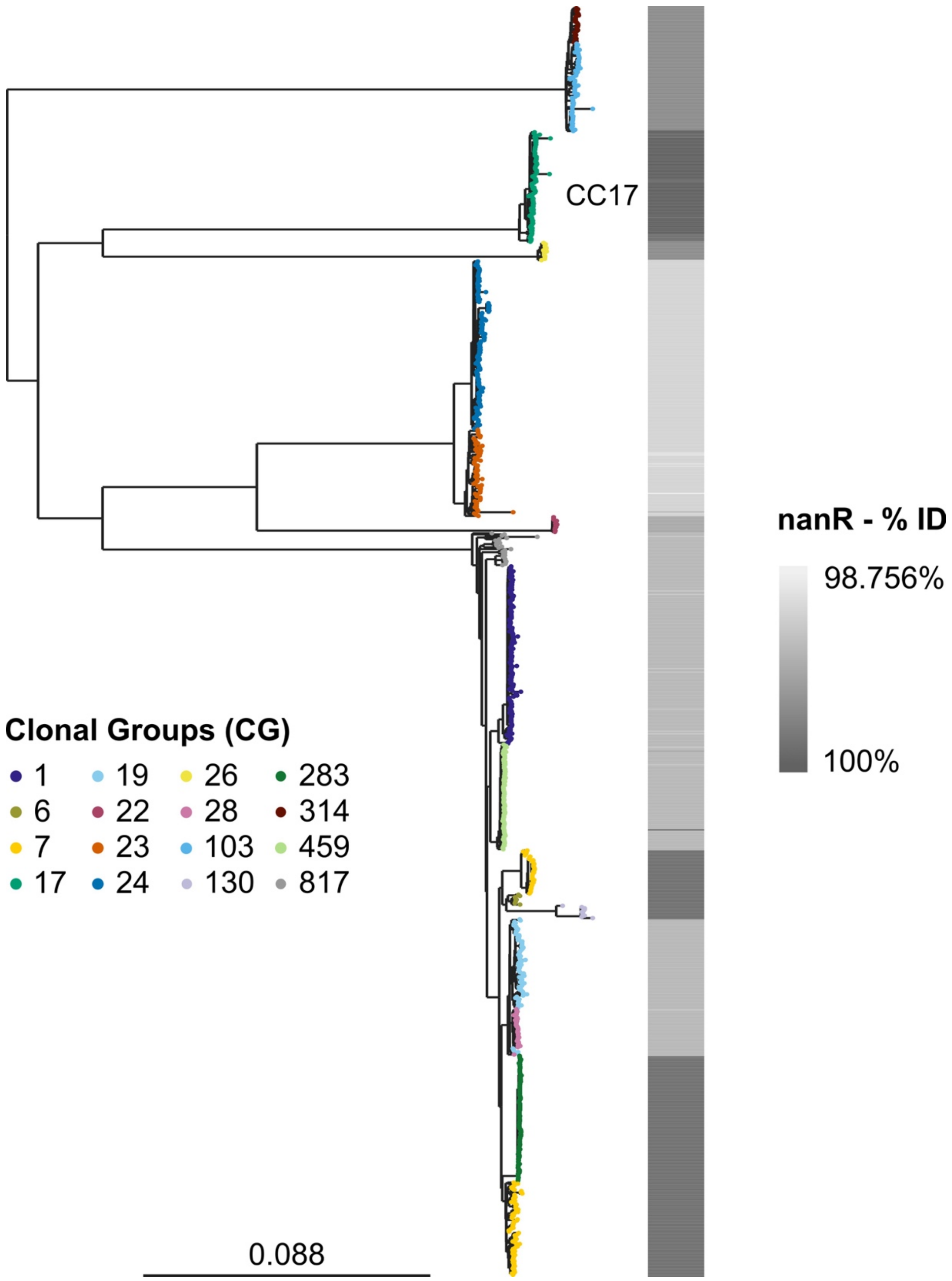


**Supplementary Figure 2: *nanR* is encoded ubiquitously amongst GBS lineages**. Maximum-likelihood phylogenetic tree of 1030 GBS core genomes for strains isolated from human, bovine and piscine hosts (adapted from(10)). Grey leaf colour indicates presence of *nanR*, and identity compared with CC17 *nanR* allele is depicted by shading. All alleles share 98.756 % identity.
